# Herbivory and Competition of Tibetan Steppe Vegetation in Winter Pasture: Effects of Livestock Exclosure and Plateau Pika Reduction

**DOI:** 10.1371/journal.pone.0132897

**Published:** 2015-07-24

**Authors:** Richard B. Harris, Wang Wenying, Badinqiuying, Andrew T. Smith, Donald J. Bedunah

**Affiliations:** 1 Department of Ecosystem and Conservation Sciences, University of Montana, Missoula, Montana, United States of America; 2 Qinghai Normal University, Xining, Qinghai, People’s Republic of China; 3 Department of Life Sciences, Arizona State University, Tempe, Arizona, United States of America; 4 Department of Forest Management, University of Montana, Missoula, Montana, United States of America; Institute of Tibetan Plateau Research, CHINA

## Abstract

Rangeland degradation has been identified as a serious concern in alpine regions of western China on the Qinghai-Tibetan plateau (QTP). Numerous government-sponsored programs have been initiated, including many that feature long-term grazing prohibitions and some that call for eliminating pastoralism altogether. As well, government programs have long favored eliminating plateau pikas (*Ochotona curzoniae*), assumed to contribute to degraded conditions. However, vegetation on the QTP evolved in the presence of herbivory, suggesting that deleterious effects from grazing are, to some extent, compensated for by reduced plant-plant competition. We examined the dynamics of common steppe ecosystem species as well as physical indicators of rangeland stress by excluding livestock and reducing pika abundance on experimental plots, and following responses for 4 years. We established 12 fenced livestock exclosures within pastures grazed during winter by local pastoralists, and removed pikas on half of these. We established paired, permanent vegetation plots within and outside exclosures and measured indices of erosion and biomass of common plant species. We observed modest restoration of physical site conditions (reduced bare soil, erosion, greater vegetation cover) with both livestock exclusion and pika reduction. As expected in areas protected from grazing, we observed a reduction in annual productivity of plant species avoided by livestock and assumed to compete poorly when protected from grazing. Contrary to expectation, we observed similar reductions in annual productivity among palatable, perennial graminoids under livestock exclusion. The dominant grass, *Stipa purpurea*, displayed evidence of density-dependent growth, suggesting that intra-specific competition exerted a regulatory effect on annual production in the absence of grazing. Complete grazing bans on winter pastures in steppe habitats on the QTP may assist in the recovery of highly eroded pastures, but may not increase annual vegetative production.

## Introduction

Livestock grazing has been the dominant land use on the rangelands of Central Asia for centuries, providing sustenance for pastoralists and products for trade. On the Qinghai-Tibetan plateau (QTP) in the People’s Republic of China, rangelands are variously reported as overgrazed or degraded, yet controversy persists regarding the extent, causes, and proposed remedies [1–4]. Within postulated human causes leading to negative trends in rangeland condition, a fundamental distinction can be drawn between livestock over-stocking arising from population growth or poor practices employed by indigenous Tibetan pastoralists on one hand, and over-stocking arising from misguided government policy on the other [5]. Within postulated biological drivers of degradation, controversy remains regarding the role of the common colonial lagomorph, the plateau (or black-lipped) pika (*Ochotona curzoniae*) [6–9]. These pikas achieve high densities where plant cover is sparse and/or short [10–14], and thus are frequently considered grassland pests [11–16]. Chinese government policy favors reducing or eliminating the species [7,8,17], but numerous studies have shown that plateau pikas play key roles on the QTP ecosystem, acting as a keystone species for biodiversity [18–23] as well as an ecosystem engineer, moderating hydrology at the local scale by increasing the rate of infiltration [24]. Although there is no dispute that global climate change has affected vegetation and hydrology on the Tibetan plateau, there is little consensus regarding site- or season-specific changes in temperature or precipitation patterns [25–28], nor on how vegetation has responded [29–37]. Unsurprisingly, recommendations for reversing negative trends in rangeland condition vary considerably [2,3,5,38–39].

Most investigations of rangeland trends have occurred at a broad scale, typically depending on remote sensing data [40–43]. Although useful and necessary, these studies may overlook important dynamics that occur on the scale of individual plant-animal interactions [3]. Wild, and later domestic, animals have been present on, and influenced vegetation on the Tibetan plateau for long enough to have affected evolutionary processes. Transhumant pastoralism involving yaks has been documented going back over 8,000 years [44–46], and a wide array of wild herbivores have always been present [12,13]. The long history of traditional pastoralism suggests that deleterious effects from grazing may, to some extent, be compensated for by reduced plant-plant competition. Both plant-plant and plant-animal dynamics are complex, however, and may vary qualitatively along environmental and density gradients [47]. Detailed, site-specific investigations can thus help policy makers gain insight into the causes, and ultimately remedies, of deteriorating rangeland condition. In particular, the relative roles of herbivory and competition in controlling vegetation response to the presence of consumers remain a central, yet poorly understood issue, on the QTP.

Because biological interactions in the field are so complex, one approach to isolating causative mechanisms is to intercede directly, typically by removing or altering one or another biological component. Although it does not represent well any naturally occurring process, the exclusion of large-bodied herbivores (livestock, in this case) can help elucidate the ways in which vegetation responds to their presence. Thus, exclosure experiments have become a common investigative technique. Recent livestock exclosure experiments on the QTP have suggested that vegetation biomass and soil nutrients have increased with protection from livestock, although not necessarily in a linear fashion [48–59].

We report here on a 5-year study in which we manipulated grazing levels of livestock and pikas. We approached our study with the recognition that rangelands vary in their history and existing management at local spatial scales, and thus that selection of numerous sites, each of which can be characterized, can provide insights unavailable from selecting only one or a few sites. We also isolated the effects of the seasonality of herbivory, in contrast to some studies on the QTP in which grazing occurred both during growing and senescent periods [48, 54, 58–59]. Most traditional grazing systems on the QTP employ seasonal grazing strategies, and grazing during the growing season may affect vegetation differently than grazing during the dormant season. This focus on seasonality also allowed us to distinguish effects of exclusion on annual production from effects on standing biomass. The distinction is important because, if livestock grazing is envisioned as continuing into the future on the site, some of the standing plant biomass must necessarily be transformed into animal biomass. Questions bearing on sustainability are more directly addressed by investigating the dynamics of plant regrowth following herbivory. Finally, we suspect that species, even those within functional groups (e.g., grasses, sedges, forbs) may respond differentially to grazing and its absence. Our field protocols allowed quantification of biomass to the genus or species level.

Our objectives were to examine patterns of annual production of QTP steppe vegetation exposed to dormant season grazing by livestock (yaks, goats, and primarily sheep, *Ovis aries*) and year-round activities of the non-hibernating plateau pikas, by altering the presence of both consumers via exclusion and removal. We monitored biomass of the most abundant plant species, as well as physical indicators of rangeland condition over 5 years (one year prior to experimental intervention, 4 years afterward) on paired plots. We employed fenced livestock exclosures and pika removal not as direct management tools, but rather as experiments to isolate herbivory and trampling from other factors, and thus to clarify their effects on site characteristics and common plant species.

## Materials and Methods

### Study Area

**Fig 1:**
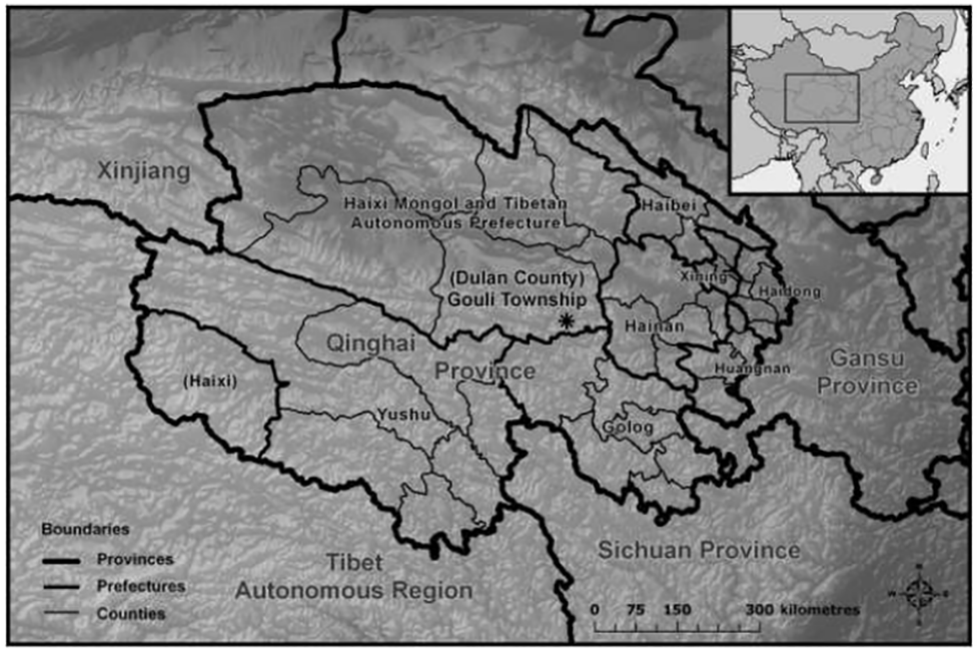
Map of Qinghai Province, China. Provincial boundary indicated by bold line, county boundaries by fine lines, and the location of Gouli Township within Dulan County with an asterisk (*). Inset shows location of Qinghai Province within the People’s Republic of China.

Our study was carried out in Village Five of Gouli Township, Dulan County, Qinghai Province, China, approximately 35.5° N, 98.7° E. (Fig 1). Village Five consisted of approximately 175 residents in 37 households, almost all of whom engaged primarily in semi-nomadic pastoralism. Distance to the nearest concentration of houses to our study area was approximately 6 km; this village was adjacent to a historic but rejuvenated Tibetan Buddhist monastery [60–61]. The landscape, part of the eastern section of the Kunlun mountain chain, was characterized by rolling hills at elevations < 4,100 m, rising to moderately-sloped peaks at ~ 4,900 m. Vegetation was sparse above approximately 4,700 m. Vegetation formations were alpine steppe (dominated by *Stipa purpurea*) at elevations < 4,300 m, alpine meadow (dominated by *Kobresia* spp.) at higher elevations, and shrublands (dominated by *Salix* spp.) on northerly exposures.

The nearest government-operated weather station was located in the county seat, Dulan, approximately 90 km (straight-line) distance and 1,000 m lower in elevation, so could not be used directly to estimate temperature or precipitation. We thus sampled temperature and precipitation for the Gouli site from interpolated meteorological data using ANUSPLIN software version 4.2 [62], which uses an algorithm based on the thin plate smoothing splines of multivariates [63–64]. Observations from 836 weather stations in China were interpolated to the 1-km spatial resolution and 8-day time step. Yearly precipitation at the study site during 2008–2013 averaged 398.0 mm (SD = 53.4), with approximately 92% falling during April through September. Mean annual temperature was approximately -1.4°C, with the warmest 8-day periods annually averaging 14.0°C and the coldest averaging -16.3°C.

The study area had been subject to international hunting focused on blue sheep (*Pseudois nayar)* until 2006 [13,65], but this activity likely had little impact on pastoral practices or vegetation. As had been common on the QTP, government-sponsored workers conducted a poisoning campaign targeting pikas during January 2007. Subsequent work showed that within a few years, pikas had repopulated most areas. The Tibetan fauna includes a number of wild ungulate species that could have foraged on experimental plots [12,13], but only the Tibetan gazelle (*Procapra picticaudata*) was ever observed in the vicinity of the experiments reported on here. In addition to plateau pikas, small mammals present in the general area included the fossorial rodent plateau zokor (*Eospalax fontanierii* [66]), the jerboa (*Allactaga sibirica*), mountain voles (*Neodon* spp.), voles (*Microtus* spp. and *Lasiopodomys* spp.), and dwarf hamsters (*Cricetulus* spp.). Himalayan marmots (*Marmota himalayana*) and Tibetan woolly hares (*Lepus oiostolus*) occurred nearby, but were never observed in the immediate vicinity of these exclosure experiments.

The entire study area was grazed by livestock and used primarily as winter pastures. Grazing generally occurred only after animals returned from summer (and sometimes autumn) pastures, generally in mid-October, until leaving for spring/summer pastures in mid-June the following year [61]. Throughout, we refer to winter grazing by the year beginning in January; thus, for example, we use the term ‘winter 2010’ to refer to grazing that occurred during ~ October 2009 through early June 2010. Village Five constituted relatively high elevation pastures within Gouli, and had been used as summer and transitional (spring-fall) pastures before the prior collective system was dismantled (which occurred in 1983 in this area). Pastoral families owned long-term leases on set pasture lands, but not all grazed their own livestock on their own pastures. Rather, many pastoralists in Gouli had begun sub-leasing their pastures to other grazers, and/or paying rental fees to graze their livestock on lands contracted to others.

Because our intent was to examine the effects of grazing practices as actually implemented by Tibetan pastoralists, responding as they wished to the physical, biological, cultural, economic, and policy environments in which they found themselves, we made no attempt to control the type or intensity of grazing [60]. Grazing pressure varied during the years prior to our study, as well as during each of the winters prior to our 5 years of summer-season measurements.

Exclosure experiments were situated in pastures grazed by 4 different Tibetan pastoralists (Table 1). Pastoralist K, who controlled land on which 3 of our experiments were placed, had recently experienced family illness, and had had to sell much of his livestock herds to pay medical expenses. At the outset of our study in 2009, K owned only 60 yaks and 25 sheep. His pasture was among the largest in Village Five (6.8 km^2^), but much of it was steep and rocky. Pastoralist S, who controlled land on which 6 of our experiments were placed, was among the wealthiest pastoralists in Village Five, but had begun engaging in a number of non-pastoral economic activities by 2009. In winter 2010, S leased his pasture to a pastoralist from Village Six to graze his herd of approximately 320 yaks. Very little winter grazing occurred on S’s pasture during winter 2011, but he leased it to other pastoralists for grazing of large (~ 300 animals) sheep herds during winters 2012 and 2013. Two of our experiments were located on the pasture controlled by pastoralist B. This relatively small (0.46 km^2^) and entirely fenced pasture had, until 2009, belonged to S, who had managed it as emergency winter reserve, and stocked it very lightly. B sub-contracted his small pasture to other local pastoralists during the winter prior to our measurements, with herds usually of about 50 sheep. The last of our experiments was located on the western boundary of the 6.2 km^2^ pasture controlled by pastoralist L, who also generally rented his pasture to others, who in turn placed large herds of both sheep and yaks on it. However, with the exception of S’s pasture during winter 2010, both observations and limited telemetry monitoring showed that yaks used high elevation pastures far from the experiments here; hereafter, we use the term “livestock” to refer exclusively to sheep and goats. We asked for and received permission for access to each of the pastures on which we worked and for the establishment of our experimental exclosures. We also worked under a letter of authorization provided by the Dulan County Forestry Bureau.

**Table 1:** Characteristics of 4 pastures on which experimental exclosures and removals were conducted.

Pasture	Pasture Size (km^2^)	Experiments	Mean Sheep/ha 2009)	Mean Elevation(m)	Mean Slope (°)
Pastoralist K	6.8	1,3,4	0.05	4,223	22
Pastoralist B	0.5	2.5		4.064	6
Pastoralist S	10.1	6–11	0.15	4.155	22
Pastoralist L	6.2	12	0.36	4,280	16

Shown are pasture size, which experiments were located on each, mean density of wintering sheep, and mean elevations and slope of the entire pastures. We were unable to estimate sheep density for Pastoralist B during the experiment.

Although Village Five contains various slopes and aspects, and mountain peaks extend to > 4,900 m in elevation, all experiments reported on here were located on gentle, generally southerly or south-easterly-facing slopes at elevations between 4,046 to 4,107 m. We generally expected to find that species preferred as forage by livestock and pikas would respond to lower herbivory pressure by increasing production relative to the grazed state. In contrast, we anticipated that plant species avoided by grazers would be more productive where grazing occurred than where grazing was restricted.

### Livestock exclosures and pika reduction

We selected 12 locations for livestock-exclusion/pika reduction experiments, based on specific hypotheses we wished to test. At each we erected livestock exclusion fences measuring 10 x 10 m using standard fencing material in use by pastoralists in western China. All fenced livestock exclosures were built in early September 2009. We re-checked exclosure fencing each summer to reduce the probability of undesired or undocumented livestock incursions.

**Fig 2:**
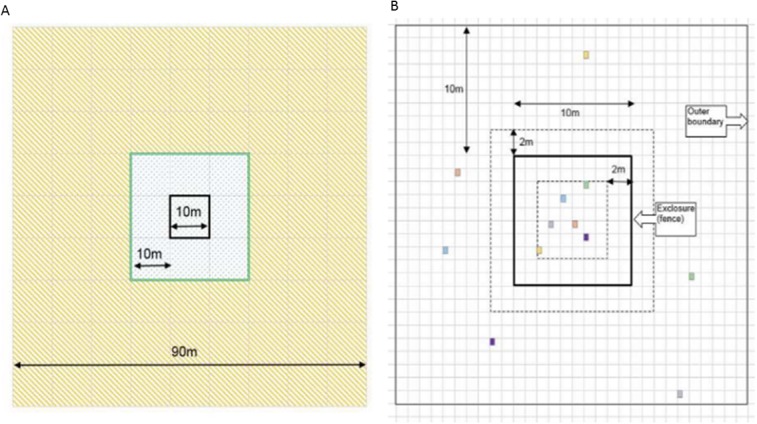
Schematic diagrams showing the experimental designs to exclude livestock and reduce pikas. A. Experimental design, showing the fence (solid black line), the area within which paired, non-exclosure plots were located (wide green line), and the 30 m extending beyond the experiment on each side (orange-hatching) within which pikas were reduced by snap-trapping during 2010–2013, Gouli Township, Qinghai Province, China. B. Schematic of exclosure experiment, showing typical placement of 12 vegetation plots established in September 2009, Village Five, Gouli Township, Qinghai Province, China. Bold solid line represents the 100 m^2^ livestock exclusion fence. Within the inner 64 m^2^ of the exclosure (dashed line), 6 vegetation plots (0.5 m^2^ each; colored squares) were randomly distributed (see text). Six paired vegetation plots that were similar in vegetation composition and density to each of the random plots (colors represent pairing) were selected within the area extending 10m in each direction from (but not within 2 m, dashed-line, of) the exclusion fence.

We selected 6 of the 12 experiments (defined as fenced-exclosures as well as the surrounding, unfenced 80 m^2^, Fig 2A), for pika reduction. Pikas were killed using commercially-available metal rodent traps placed adjacent to all active pika burrows, both within and outside these fenced exclosures. To reduce incursion and repopulation of pikas outside the experiment, we extended our trapping beyond the 10-m wide off-exclosure strips by an additional 30-m, so that the total area subjected to pika reduction measured approximately 8,100m^2^, Fig 2A. The protocol for our study was approved by the Institutional Animal Care and Use Committee at Arizona State University (Protocol #12-1231R), as well as the Dulan County Forestry Bureau, Dulan, Qinghai, PRC.

### Experimental design

Because exclosure experiments were not randomly selected from within Village Five but rather were chosen to represent a variety of initial conditions, we did not consider them as random effects. Instead, we treated exclosure experiments independently, grouping them when appropriate to test specific hypotheses. Experiments 1 and 2 were adjacent to one another, separated by a preexisting fence that demarcated the boundary between pastures managed by pastoralists B and K. Experiment 1 (in pasture belonging to K) was located just above a seasonal water-course, an area which we had noted served as a frequently-used livestock pathway. This area was characterized by a relatively high percent of bare soil and low vegetation cover at the experiment’s outset (Table 2). The pasture managed by pastoralist B, in which experiment 2 was located, had for a number of years prior to our study served as an emergency-winter pasture and was rarely used by livestock (reflected in the relatively high vegetation cover and little bare soil, Table 2). We reduced the abundance of pikas in and around both experiments beginning in summer 2010. We were particularly interested in examining the influence of past management and the livestock trailing route on otherwise similar vegetation.

**Table 2:** Initial conditions at each of the 12 experiments.

	Pika	Veg%	Soil	Ersn	Lt%	*Cata*	*Carx*	*Heal*	*Kobr*	*Lese*	*Oxyt*	*Poa*	*Pobi*	*Stpu*	*Thla*	*Bio*
1	34.5	52.1	47.0	5.0	0.9	12.3	0.0	12.8	0.6	0.1	0.2	0.0	0.2	91.2	0.0	124.0
	*13*.*1*	*11*.*4*	*11*.*2*	*0*.*0*	*0*.*5*	*17*.*0*		*13*.*2*	*0*.*8*	*0*.*5*	*0*.*6*		*0*.*6*	*33*.*0*		
2	11.0	85.4	12.6	2.6	1.9	3.3	0.0	7.4	6.6	1.6	0.1	1.4	0.0	302.7	0.0	328.5
	*4*.*2*	*6*.*2*	*6*.*0*	*0*.*8*	*1*.*9*	*3*.*7*		*13*.*5*	*3*.*0*	*2*.*7*	*0*.*3*	*1*.*6*		*95*.*0*		
3	37.5	81.0	15.2	2.0	3.8	11.5	0.0	4.3	8.4	1.6	12.0	11.3	12.3	114.7	56.6	232.9
	*3*.*7*	*7*.*0*	*6*.*0*	*0*.*5*	*1*.*8*	*26*.*8*		*5*.*5*	*4*.*5*	*2*.*6*	*8*.*1*	*6*.*0*	*5*.*7*	*19*.*6*	*50*.*9*	
4	82.0	70.5	25.9	2.7	3.6	7.1	0.0	0.5	8.3	11.4	1.6	1.2	20.1	51.8	56.2	160.0
	*28*.*4*	*21*.*3*	*20*.*2*	*1*.*2*	*2*.*6*	*12*.*6*		*0*.*8*	*9*.*6*	*7*.*4*	*2*.*0*	*1*.*3*	*14*.*9*	*13*.*7*	*34*.*6*	
5	46.4	71.0	22.5	2.0	6.3	0.0	3.0	0.0	3.8	5.3	0.4	6.9	1.1	93.4	17.3	131.7
	*4*.*6*	*10*.*7*	*9*.*0*	*0*.*5*	*4*.*3*		*3*.*6*		*2*.*9*	*6*.*0*	*0*.*5*	*3*.*4*	*1*.*5*	*20*.*3*	*16*.*0*	
6	31.0	69.2	25.0	3.2	5.9	0.0	0.0	0.0	5.4	5.4	0.0	6.7	1.2	101.8	0.0	120.6
	*6*.*0*	*6*.*8*	*7*.*2*	*0*.*5*	*3*.*5*				*1*.*2*	*2*.*5*		*3*.*3*	*1*.*9*	*20*.*7*		
7	38.0	60.0	37.5	4.8	2.5	7.1	0.0	56.7	0.3	0.2	0.0	0.0	1.3	92.3	56.4	232.2
	*2*.*1*	*6*.*4*	*5*.*8*	*0*.*5*	*1*.*4*	*7*.*0*		*16*.*2*	*0*.*5*	*0*.*3*			*1*.*8*	*18*.*0*	*48*.*0*	
8	30.0	56.7	38.3	3.5	4.6	0.9	0.0	0.0	5.8	1.2	0.1	1.6	0.0	97.2	58.9	166.8
	*7*.*3*	*6*.*9*	*6*.*0*	*0*.*5*	*1*.*6*	*1*.*2*			*1*.*7*	*1*.*8*	*0*.*1*	*1*.*4*		*20*.*6*	*39*.*8*	
9	24.5	67.1	28.2	1.8	4.8	1.6	0.0	37.4	7.3	0.0	0.0	0.7	0.0	67.8	0.0	114.9
	*1*.*6*	*5*.*8*	*6*.*0*	*1*.*0*	*1*.*5*	*1*.*5*		*17*.*0*	*2*.*8*			*1*.*1*		*14*.*6*		
10	28.5	66.3	30.9	2.7	2.7	15.5	0.0	60.7	3.6	0.4	0.0	0.8	0.0	67.3	0.0	149.1
	*0*.*5*	*15*.*4*	*15*.*3*	*0*.*8*	*1*.*6*	*20*.*8*		*32*.*8*	*2*.*0*	*0*.*9*		*1*.*3*		*25*.*4*		
11	7.5	59.2	36.1	2.7	4.8	0.6	0.0	48.0	3.4	0.0	0.2	5.1	11.3	72.8	0.0	143.5
	*1*.*6*	*6*.*7*	*6*.*9*	*0*.*8*	*2*.*2*	*1*.*2*		*28*.*8*	*1*.*2*		*0*.*3*	*2*.*3*	*7*.*7*	*18*.*3*		
12	20.4	55.5	41.8	3.6	2.7	1.8	0.0	18.3	1.9	0.0	0.0	1.8	0.9	155.8	0.0	185.5
	*1*.*6*	*16*.*3*	*16*.*0*	*0*.*5*	*1*.*1*	*5*.*4*		*21*.*7*	*1*.*7*			*2*.*0*	*0*.*7*	*81*.*7*		
$\bar{x}$	32.6	66.2	30.1	3.0	3.7	5.1	0.3	20.5	4.6	2.3	1.2	3.1	4.0	109.1	20.5	174.1

Shown in Table are the experiment number (Ex), ika index (Pika), total vegetation percent cover (Veg%), percent bare soil cover (Soil), Erosion index (Ersn). percent litter cover (Ltr%), and fresh biomass (g/m^2^) of plant species meeting minimum sample size thresholds, for 12 *experiments (Cardimine tangutorum = Cata*, *Carex spp*. *= Carx*, *Heteroppapus altaica = Heal*, *Kobresia* spp. = *Kobr*, *Leymus secalinus* = *Lesa*, *Oxytropis* spp. = *Oxy*t, *Poa* spp., *Potentilla bifurca* = *Pobi*, *Stipa purpurea*, = *Stpu*, *Thermopsis lanceolata* = *Thla*, total fresh biomass = Bio), Gouli township, Qinghai Province, China, in September 2009 (i.e., before treatments). For each experiment, top row gives mean, bottom row (italics) gives SD (in all cases, *n* = 12).

Experiments 3 and 4 were both located in K’s pasture, approximately 266 m apart, and were designed to examine the effect of pika reduction. Pikas were trapped beginning summer 2010 in and around experiment 3 but not experiment 4. Experiment 4 was characterized by rocky soil and a low proportion of palatable graminoids. Both experiments 3 and 4 also included substantial amounts of *Thermopsis lanceolata*, which both our observations and existing literature had suggested was adapted to soil disturbance. We wished to examine whether protection from grazing would allow other species to gain at its expense.

Experiments 5 and 6 were adjacent to one another, separated by a pre-existing fence demarcating the boundary between pastures managed by pastoralists B and S. Here, we were interested in examining if the recent few years of very light grazing in B’s pasture had influence beyond those years when both were protected from grazing.

Experiments 7 and 8 were both located in S’s pasture, approximately 300 m apart; pikas were reduced in experiment 7, but not 8. As in the paired experiments 3 and 4, *T*. *lanceolata* appeared to be competing with *S*. *purpurea*. We hypothesized that livestock exclusion would favor species less reliant on disturbance than *T*. *lanceolata*.

Experiments 9 and 10, both in pastures managed by S, were adjacent to one another, separated by a preexisting fence demarcating S’s winter reserve (i.e., lightly grazed) area. We trapped pikas beginning in 2010 in both experiments 9 and 10. Experiments 11 and 12 were adjacent to one another, separated by a preexisting fence demarcating L’s pasture (experiment 12) from S’s pasture.

Livestock present in each pasture during winter were estimated by direct counts made by our year-round technicians, as well as interviews with wintering pastoralists. Diets of yaks, sheep, goats, pikas, and Tibetan gazelles were estimated from fecal samples collected during winter 2010. At bedding sites, we collected 4 yak samples (from the herd using S’s pasture in winter 2010), as well as 4 samples of sheep and 3 of goats at different bedding sites. Because sheep and goats were grazed together, and most herds in Gouli consisted of approximately 15% goats, we summarized diets by weighting the mean of sheep and goat diets by the ratio 85:15. During the same winter, we collected fecal pellets of plateau pikas and Tibetan gazelles opportunistically in the field. Diets were estimated as percent density using micro-histological fragment analysis at the Wildlife and Habitat Nutrition Laboratory at Washington State University, Pullman, WA, USA. The timing of our vegetation sampling, in mid-summer, precluded us from estimating consumption rates of either livestock or pikas.

### Plot selection and vegetation sampling

Within each of the 12 exclosures, we randomly selected 6 square plots measuring 0.5 m^2^ for repeated vegetation sampling. These permanent plots were selected by first demarcating each 10 x 10 m exclosure into 100 1 m^2^ sections. We excluded the outermost 2 m-wide strips of each exclosure from the sampling universe to reduce any possible edge effect caused by proximity to the fence. We numbered the remaining 64 m^2^ sequentially, and used the pseudo-random number generator in MS-Excel to generate a list of integers, 1–64. The first 6 integers on the random list for each exclosure were chosen, and the 1-m^2^ grid associated with that integer was selected for sampling (Fig 2B). We placed a square PVC plot frame measuring 0.5 m^2^ on the northeast corner of each selected 1-m^2^ section, and marked the corners with ~ 5 mm diameter steel cable anchors that were sunk approximately 30 cm into the ground, leaving a small (~3 cm diameter) loop protruding to which we attached a metal, numbered tag. In subsequent years, field crews carried the PVC frames to each plot and, upon locating the cables, fixed the frame corners to the 2 loops. In this way, we were able to re-locate each permanent plot while leaving very little physical sign or interference.

After the pre-exclosure vegetation sampling of each plot was completed (in early September 2009), we located a corresponding 0.5 m^2^ area, outside of, but no nearer than 2 m or further than 10 m from the exclosure fence to act as its pair. We required that the paired plot be located at approximately the same slope, elevation and aspect as the in-exclosure plot, and that it contain the same species mix and in similar abundances. These outside-exclosure plots were considered that particular within-exclosure plot’s pair (Fig 2B), and subsequent analysis focused on the differences in vegetative measurements between the two. Thus, our total sample size consisted of 6 0.5 m^2^ plots both inside and outside each of the 12 exclosures (*n* = 144 plots/year), during both the pre-exclosure year (2009) and the subsequent 4 summers (2010–2013), i.e., total *n* = 720 plot readings. Plots were sampled in early September 2009, mid-September 2010, mid-September 2011, mid-July 2012, and late July 2013. Because livestock grazing on distantly-located summer pastures began in June, there was little opportunity for vegetation produced in the current year to be lost to ungulate herbivory, and thus our sampling represented annual production.

Vegetation data were collected by crews of trilingual (Tibetan, Chinese, English) seasonal technicians. Prior to each field season, crews were trained in species identification and field protocols. Field crews quantified plant species presence, height, cover, and current-year fresh biomass for each species in each plot, as well as ground cover estimates of total vegetation, bare soil and litter (previous years’ dry vegetation). Above-ground biomass was estimated for each species in a step-wise process. First, crew members standardized their estimations of species-specific fresh-weight by collecting reference samples of known fresh-weight (usually 1 g) from an adjacent, off-site location. Species-specific fresh biomass estimates were estimated by moving the known-weight reference sample within the plot, counting the number of similarly-sized plant clusters of each species. Second, to provide on-going quality control of fresh-weight estimation, a system of random check-plots was set up, in which crews did not know until after collecting all vegetation data at a given plot whether that plot had been selected for calibration. If selected, a nearby 0.5m^2^ location with similar vegetation to the plot was identified, subjected to the full measurement protocol, and then clipped and sorted to species. Species-specific fresh-weights of the check-plot were recorded, and compared with the actual (non-clipped) plot. At each plot, an index of pika burrow density (“pika index”, hereafter) was also obtained by counting all active burrows in a 7-m radius (154 m^2^) around the plot center.

We quantified species-specific annual production as fresh biomass (g) for each plant species with a mean of >1% cover averaged over all plots within the experiment in the pre-treatment year 2009 (Table 2). In addition to species-specific fresh weight, we examined the 4 metrics “Litter cover (%),” “Bare soil (%),” “Erosion index” and “Total live vegetation cover (%).” For erosion index, we used an ordinal scale 0 = none, 1 = low, 2 = low-moderate, 3 = moderate, 4 = moderate-high, 5 = high, 6 = severe, based on characteristics such as rills, gullies, and pedestalling [67]. We assessed the effectiveness of pika reduction by visually counting individual pikas seen within the livestock exclosure, as well as one randomly-selected 100 m^2^ patch adjacent to each exclosure (both those with pikas reduced and uncontrolled) during 1-hour observation bouts during summers 2010 through 2013.

### Hypotheses tested and statistical analyses

Following our experimental design, our approach to analysis was to look first at our planned 2-way experiment-wise comparisons (described above under “Experimental Design*”*). Where significance tests indicated that experiments differed and/or interaction terms were significant, we examined individual exclosures, as described below. We conducted further tests on experiments grouped within the pasture that contained each. Because our attempts to reduce pika abundance in some cases resulted in only a modest difference in resultant abundance among experiments designed as treatment vs. control, we also examined selected *a posteriori* contrasts among experiments with the most divergent pika abundances post-reduction.

To address hypotheses that livestock exclusion affected vegetation, we used mixed-effects linear models, with raw paired-plot differences (defined as the value of the within-exclosure plot minus the value of the outside-exclosure plot) as response variables, within-exclosure plots as random explanatory effects, and “treatment” (coding year 2009 as pre-treatment and years 2010–2013 as post-treatment) and experiment (where more than one entered the model, see below) as a fixed, explanatory factors. Positive values indicated greater abundance within the exclosure than in the paired plot outside the exclosure. Recognizing that a binary characterization of pre- or post-treatment could fail to capture vegetation trends post-treatment, we also used mixed-effect linear models to assess the trend of differences from the pre-exclosure condition (scaled such that all paired-plot differences in 2009 were set to zero) on time during years 2010–2013. In these models, we again considered paired-plot differences as response variables, exclosure plots as random explanatory effects, and experiment (where appropriate) and year-since-treatment as fixed, explanatory effects. Here, we interpreted the slope of the response variable with time as the primary metric of interest, and the intercept of that same relationship as the initial response in year 2010 (i.e., the null hypothesis was that the intercept in year 1 was zero).

To address hypotheses that pika reduction affected vegetation, we were unable to use the same design as above because we had no way to select paired plots within each experiment. The scale on which we needed to trap pikas (8,100 m^2^) to produce effective reduction on each experiment left us no choice but to make comparisons among (rather than within) experiments. Thus, we used raw values of all plot measurements (rather than differences among plots paired *a priori*) within experiments that differed primarily in whether or not pikas were reduced. We tested hypotheses that pika reduction explained observed differences by examining the interaction of treatment on experiment (where treatment was coded as years before and after pika reduction began), with the fixed effect being the treatment, and considering plot number as random effects. That is, pika reduction was interpreted has having had an effect when significant year-on-year differences corresponding with the initiation of pika removal were themselves dependent on whether the differences among the experiments (pikas reduced vs. not) were also significant. Our analyses pooled data from within exclosures (i.e., livestock excluded and pikas reduced), and outside exclosures (grazed by livestock and pikas reduced); thus, we also examined these 2^nd^ order interactions.

Temperature, precipitation and other weather factors varied annually, and measurements were not taken on exactly the same date (and thus phenological stage) each year. As well, fresh-weight biomass likely varied by species, year, and date of sampling (we lacked sufficient data on all species to use dry-weights). However, because our design focused on differences among paired-plots that varied only by the intensity of livestock and pika herbivory, because paired plots were located a maximum of 20 m from one another on similar slopes and aspects, and because vegetation on paired-plots was always measured on the same day by the same field crew using the same sampling methods, we effectively controlled for all such variation.

To account for the multiple comparisons inherent in our approach, we used *P* = 0.025 as a marker for significance, accepting a 1 of 40 probability that comparisons we accepted as true differences arose merely by chance. For brevity, we present results only from tests reaching this threshold. All statistical tests were conducted using JMP 11.1.1 (SAS Institute, Cary, N.C., USA).

## Results

### Initial conditions and the effectiveness of livestock exclosure and pika reduction

Mean live vegetation cover prior to livestock exclusion over all experiments was 66.2%, and varied from 52.1% to 81.0%. Mean litter cover over all experiments was 3.7%, varying from 0.9 to 6.3%. We documented a total of 26 plant species within experimental plots, but focus hereafter on the 10 that were found in greater than trace amounts (Table 2). *Cardamine tangutorum*, *Kobresia* spp., and *Stipa purpurea* occurred on all 12 experiments. Vegetation at the onset of the experiment was dominated by *S*. *purpurea*, overall constituting almost 63% of all live biomass. The 2^nd^ most abundant species was *Heteropappus altaicus*, occurring on 9 experiments. *Thermopsis lanceolata* occurred on only 5 experiments, but was abundant when present, and was the 3^rd^ most abundant species by fresh biomass. Fresh biomass over all experiments averaged 174 g/m^2^ at the onset of the experiment, varying from 115 to 328 g/m^2^.

**Fig 3:**
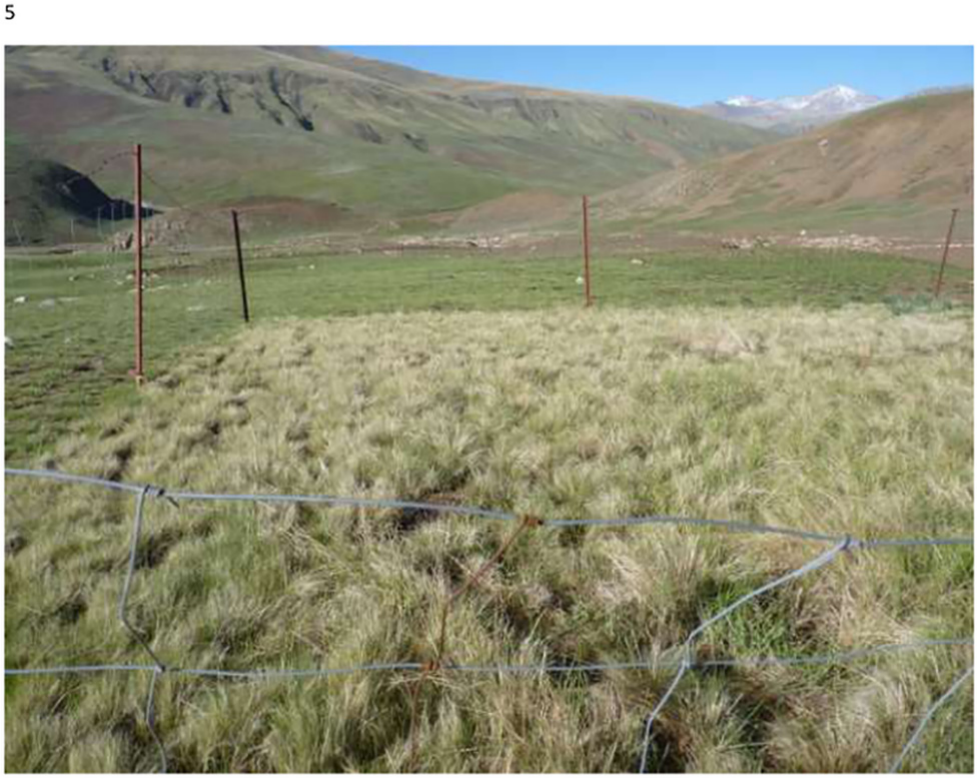
Experiment 3, shown in July 2010. Visible are the larger amounts of litter, primarily of *Stipa purpurea*, within the livestock exclosure than outside. Also evident is the larger amount of live *S*. *purpurea* inside than outside. In this experiment, *S*. *purpurea* fresh biomass increased the first year following exclosure, but subsequently declined to levels similar to or below those outside the exclosure.

We encountered only a single instance in which livestock had evidently damaged the exclosure fence and gained access (exclosure 1 in summer 2010). However based on the small amount of physical evidence (few feces and little evidence of grazing), we elected to repair the fence and retain the exclosure within the experiment. We assumed that fences similarly prevented access by Tibetan gazelles, but their total biomass on the landscape was sufficiently low that we ignored any possible effects of their exclusion. Visual evidence of livestock exclusion via fences constructed in September 2009 was striking as early as July 2010 (Fig 3). Livestock density varied during the 4 winters during which livestock affected vegetation following initiation of our experiments. Our estimated mean sheep density during winters 2009–2011 was pastoralist K (experiments 1,3,4) ~ 0.05 sheep/ha, pastoralist S (experiments 6–11) ~ 0.15 sheep/ha plus another ~ 0.15 yak/ha (only in winter 2009–10), and pastoralist L (experiment 12)~ 0.36 sheep/ha. We were unable to quantify sheep density for Pastoralist B (experiments 2, 5) but it appeared intermediate between that of Pastoralists K and S.

Our pika index prior to both livestock exclusion and pika reduction varied from 7.5 to 82.0. In the 6 experiments designed to quantify effects of pika reduction, we lethally removed at least 196 pikas (35 in 2010, 128 in 2011, 33 in 2012; Table 3). Pika removal did not result in their elimination from reduction plots, but roughly halved their density ($\bar{x}=9.04$ pikas seen/experiment/year in unreduced experiments, $\bar{x}=4.58$ pikas seen/experiment/year in reduced experiments; generalized linear models assuming Poisson distribution *F* = 9.07; df = 1,43; *P* = 0.0043).

**Table 3:** Pikas removed and observed at each experiment.

Pikas removed						Pikas observed				
	Year	2010	2011	2012	Total	2010	2011	2012	2013	Total
Experiment	Pastoralist									
*1*	*K*	*10*	*18*	*8*	*36*	*2*	*3*	*2*	*2*	*9*
*2*	*B*	*2*	*19*	*4*	*25*	*4*	*3*	*3*	*2*	*12*
*3*	*K*	*20*	*39*	*9*	*68*	*5*	*9*	*5*	*7*	*26*
4	K	0	0	0	0	15	32	9	12	68
5	B	0	0	0	0	1	12	9	10	32
6	S	0	0	0	0	3	13	2	2	20
*7*	*S*	*1*	*31*	*3*	*35*	*0*	*12*	*3*	*6*	*21*
8	S	0	0	0	0	6	15	3	6	30
*9*	*S*	*0*	*8*	*5*	*13*	*12*	*1*	*0*	*0*	*13*
*10*	*S*	*2*	*13*	*4*	*19*	*10*	*7*	*8*	*4*	*29*
11	S	0	0	0	0	2	17	2	6	27
12	L	0	0	0	0	3	22	3	12	40

Experiments in dark, italic font were subject to pika reduction during 2010–2012.

### Herbivore Diets

Approximately two-thirds of sheep diets consisted of grasses. Sheep evidently sought out *Poa*; although making up less than 2% (by fresh biomass) among commonly encountered species in our experiments, *Poa* constituted almost 24% (by frequency of occurrence) of sheep diets (Table 4). *S*. *purpurea* was the 2^nd^ most abundant species in sheep diets (15.7%), followed by *Elymus* spp. and *Leymus secalinus*. Sedges of the genera *Carex* spp. and *Kobresia* spp. made up just under 11% of sheep diets. Forbs made up the remainder of sheep diets, but we found no evidence that sheep in winter consumed any *Heteropappus* or *T*. *lanceolata* they found. Pikas in winter ate a higher proportion of sedges (47.6%) than grasses (38.3%). Tibetan gazelles ate primarily dicots even during winter (Table 4), including species often considered unpalatable to livestock such as from the genera *Artemisia*, *Oxytropis*, and *Ephedra*.

**Table 4:** Mean winter diets of livestock and wildlife.

	Sheep/Goat	Plateau pika	Tibetan Gazelle
*n*	*8*	*12*	*2*
**Grasses**			
*Elymus*	8.7		1.0
*Festuca*	1.3		
*Leymus*	7.6	11.0	
*Poa*	23.8	4.3	4.0
*Roegneria*	2.7		
*Stipa*	15.7	20.0	
*Deschampsia*	5.6		
Other Grass	1.3	3.0	2.0
**Sedges**			
*Carex*	7.6	47.6	4.0
*Kobresia*	3.3		
**Dicots**			
*Artemisia*	2.8		34.6
*Astragalus*	1.4		0.5
*Chenopodium*	1.0		
*Ephedra*	0.3		10.5
*Galium*	0.2	0.4	
*Krascheninnikovia*	8.0		
*Oxytropis*	3.0	5.9	29.0
*Pedicularis*	0.9		
*Polygonum*	0.2		3.5
*Potentilla*	0.7	2.5	4.5
*Saussurea*	0.3		
Other Forb	1.2	4.9	2.9

Data were collected at village Five, Gouli Township, Dulan County, China, January-March 2010; values estimated from fecal micro-histological analysis to genus level. Entries are the mean percent frequency, where each sample was generated from presence of the species on a 10 x 10 grid superimposed on the microscope slide.

### Effects of excluding livestock

#### Biophysical variables

**Fig 4:**
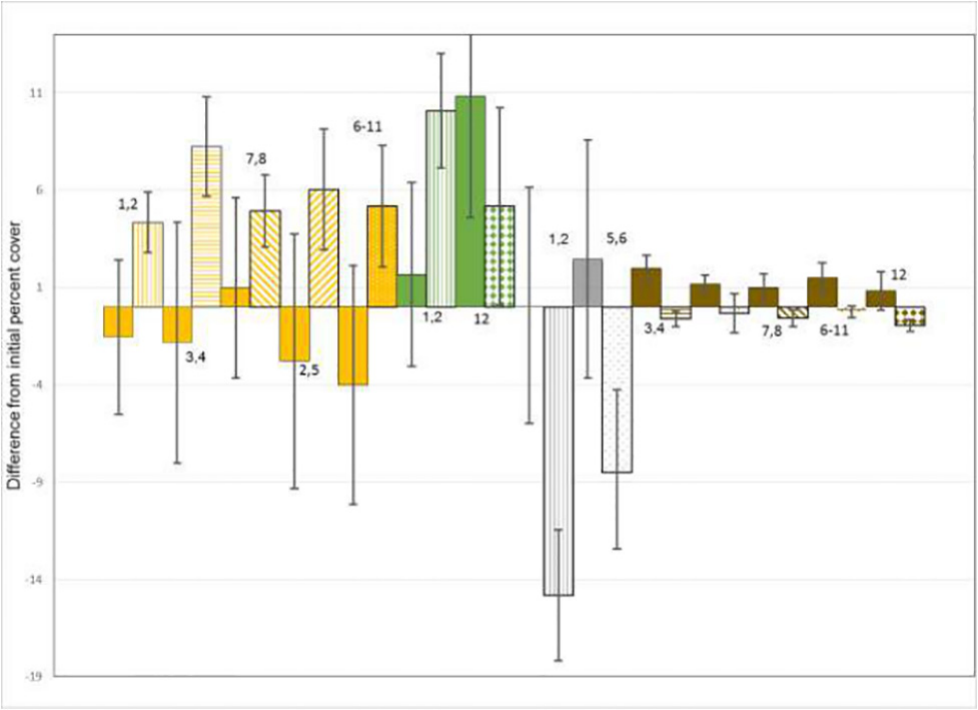
Histograms showing effects of livestock exclosure on biophysical variables in experiments where significant (*P* < 0.025). Shown are mean and 95% confidence intervals (error bars) for exclosure-caused differences in percent plot covered by litter (orange), live vegetation (dark green), erosion index (gray), and bare soil (brown). Values prior to livestock exclosure indicated by solid shading, values after livestock exclosure indicated by vertical hatching (experiments 1 and 2), horizontal hatching (experiments 3 and 4), sparse points (experiments 5 and 6), diagonal upper left to lower right hatching (experiments 7 and 8), diagonal upper right to lower left hatching (experiments 2 and 5), reverse color sparse points (experiments 6 through 11), bricks (experiment 9), and diamonds (experiment 12).

**Fig 5:**
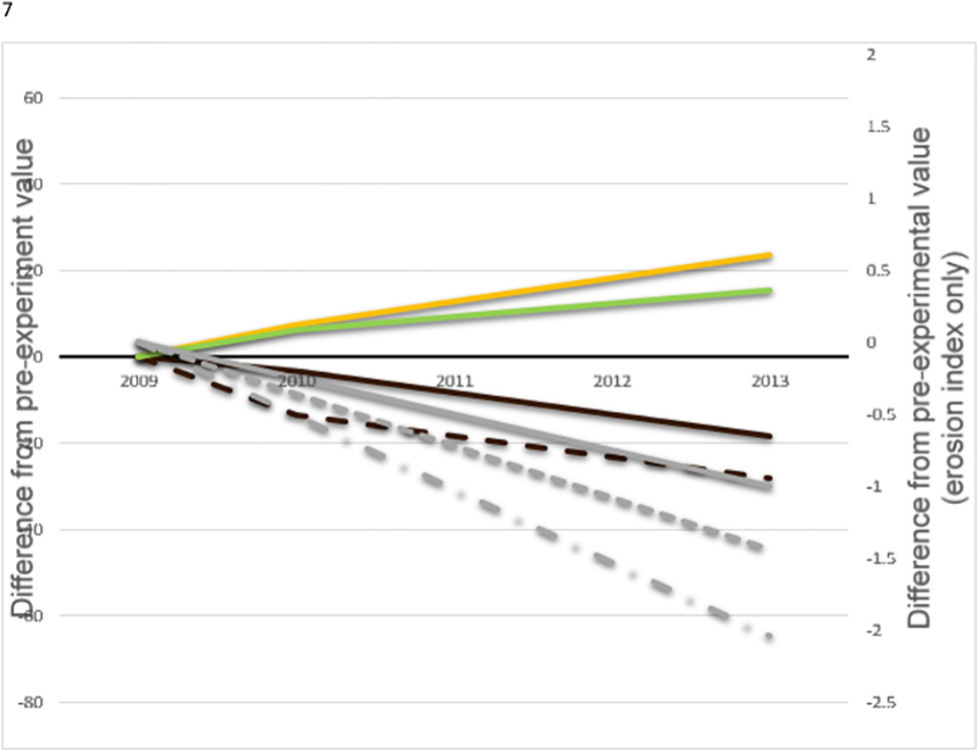
Trends on time, showing effects of livestock exclosure on biophysical variables where significant (*P* < 0.025). Shown are mean differences between within and outside livestock exclosure fences of litter (orange), live vegetation (dark green), bare soil (brown), and erosion index (gray), with all values calibrated to zero difference in 2009, the year exclosures were established. Individual experiments are indicated by solid (experiments 1 and 3), dashed (experiments 5 and 6), and dash-dot (experiment 3) lines.

Livestock exclusion increased the percentage of ground covered by litter in almost all experiments (Fig 4), and in experiment 3, litter cover significantly increased with time since exclosure. In most cases however, litter cover did not continue to increase with time relative to grazed plots. Percent cover of plots consisting of bare soil declined after livestock exclosure in experiments 1, 2, 5, and 6 and continued to decline with time since exclosure in experiments 1, 3, and 6 (Fig 5); it was not significantly affected in the other experiments. Our erosion index was reduced by exclusion of livestock in half of all experiments (Fig 4); it did not differ significantly in the other half. Additionally, erosion indices continued to decline with time in experiments 3, 5, and 6 (Fig 5). However, we found no consistent pattern in the percentage of ground cover made up by live vegetation. Livestock exclusion led to increased live vegetation cover in experiments 1 and 2, but the reverse pattern was observed in experiments 11 and 12. Live vegetation within exclosures continued to increase in cover with time relative to paired grazed plots in experiments 1 and 3 (Fig 5). No significant differences were observed in other experiments.

### Plant species

**Fig 6:**
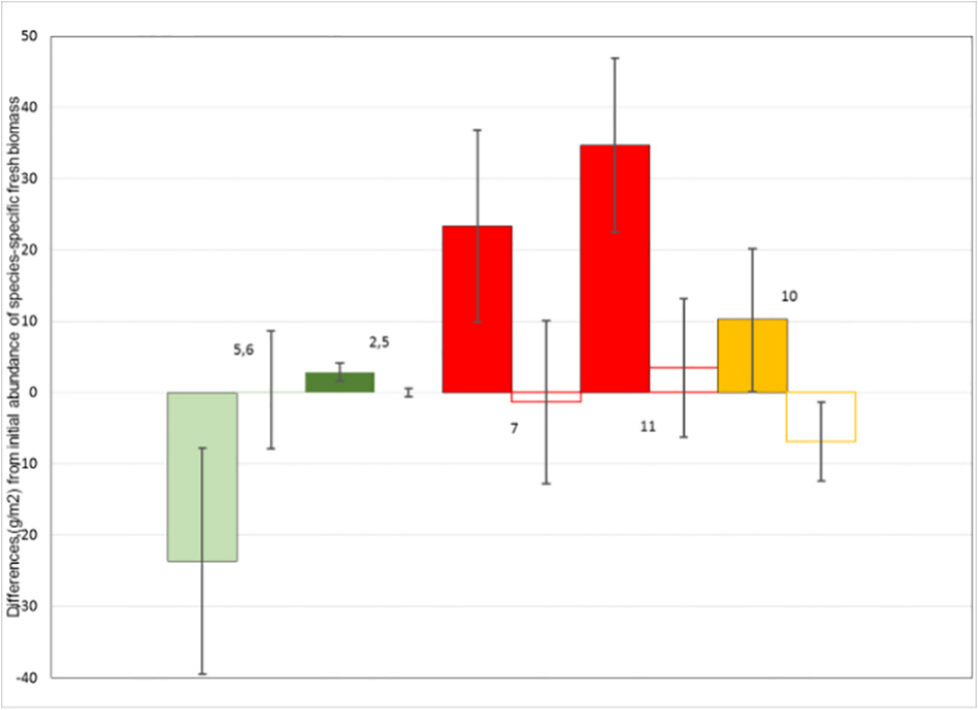
Histograms showing effects of livestock exclosure on vegetation in experiments where significant (*P* < 0.025). Shown are mean and 95% confidence intervals (error bars) for exclosure-caused differences in fresh biomass (g/m^2^) of *Stipa purpurea* (light green), *Kobresia* (dark green), *Heteropappus altaicus* (red), and *Cardamine tangutorum* (orange). Values prior to livestock exclosure indicated by solid shading, values after livestock exclosure indicated by open bars (experiments indicated adjacent to bar pairs).

**Fig 7:**
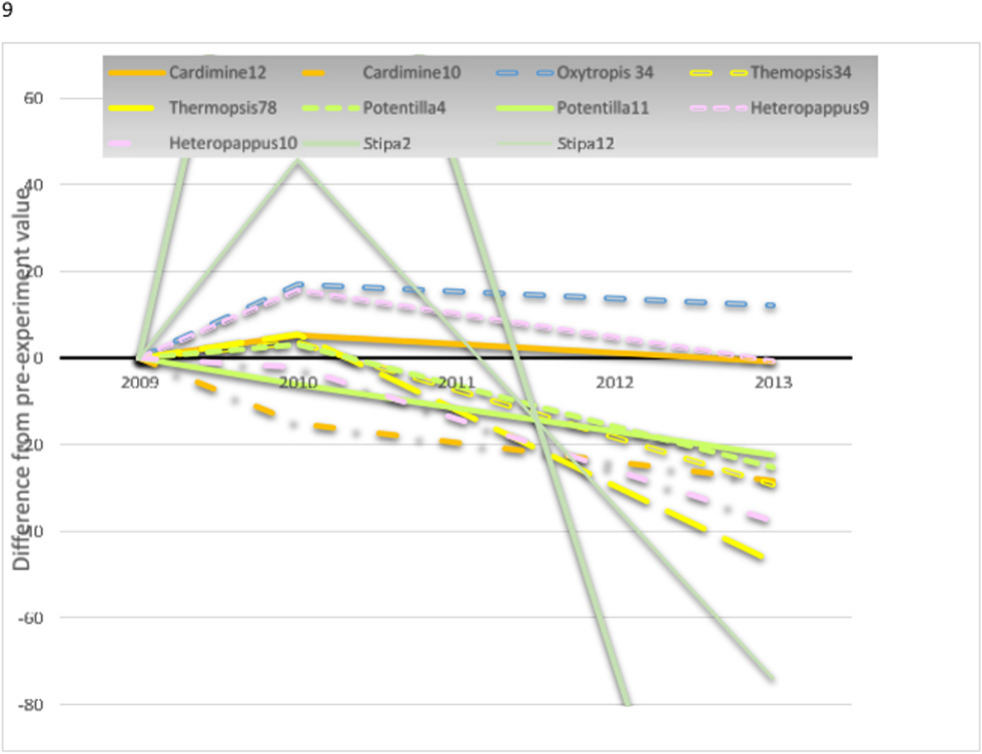
Trends on time, showing effects of livestock exclosure on plant species where significant. *Stipa purpurea* (light green), *Heteropappus altaicus* (lavender), *Cardamine tangutorum* (orange), *Thermopsis* (yellow), *Potentilla bifurca* (lime), and *Oxytropis* spp. (blue). Shown are mean differences between within and outside livestock exclosure fences, with all values calibrated to zero difference in 2009, the year exclosures were established. Individual experiments are indicated by slender solid (2), compound dash (3 and 4), short dash (4 only), long dash (7 and 8), compound short dash (9), dash-dot (10), and thick solid (12) lines.

We found more significant declines than increases in species-specific fresh biomass following livestock exclosure. *S*. *purpurea* increased following exclosure in experiments 5 and 6 (Fig 6), but displayed significantly negative trends with time since exclosure in experiments 2 and 12 (Fig 7). *Kobresia* spp., *H*. *altaicus*, and *Cardamine tangutorum* produced less fresh biomass in exclosures 2 and 5, 7 and 11, and 10, respectively, following exclosure than before (Fig 6). *H*. *altaicus* continued to decline with time since exclosure in experiments 9 and 10; *P*. *bifurca* declined with time since exclosure in experiments 4 and 11, and *T*. *lanceolata* declined with time since exclosure in experiments 3, 4, 7, and 8 (Fig 7). *Oxytropis* spp., surprisingly, was the only species to show a positive response to livestock exclusion while also failing to display a negative response (Fig 7)

**Fig 8:**
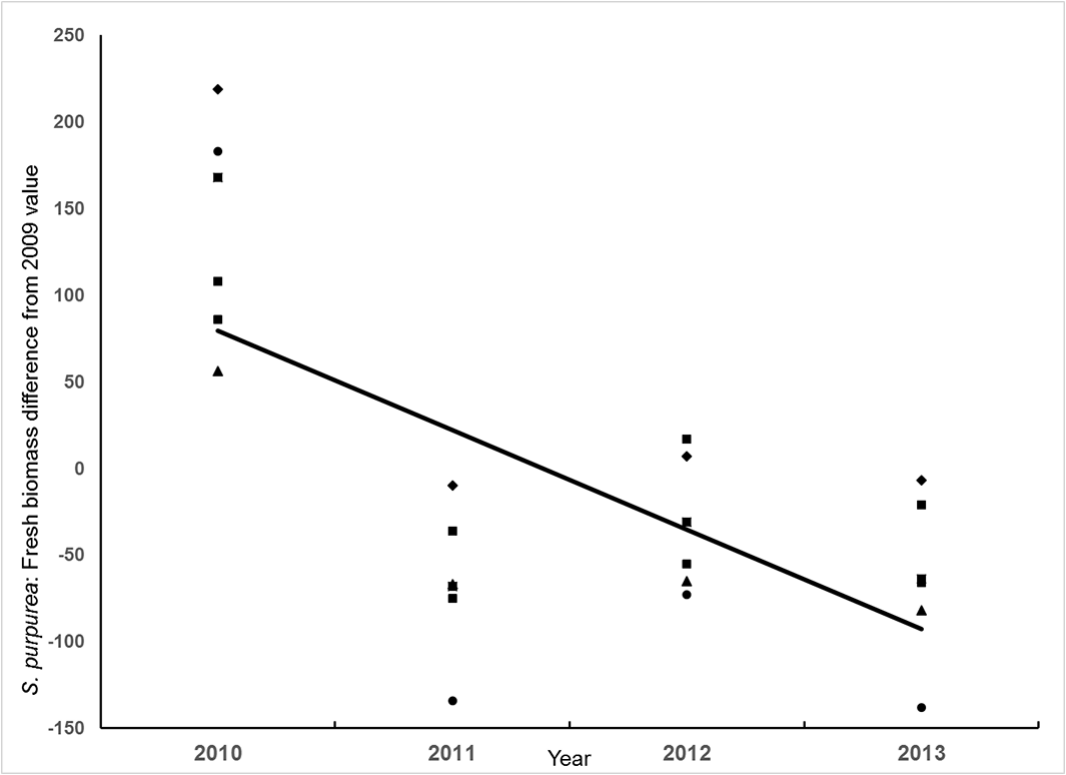
Mixed-effect linear model showing the pattern of paired-plot differences in *Stipa purpurea* fresh biomass in experiment 2. Raw data are scaled so that paired-plot differences in the baseline year 2009 were all set to zero, and vegetation plots (*n* = 6, solid circles) were treated as random effects. Y-axis scale is paired-plot differences in g/0.5 m^2^. Values in 2010 (year 0) were significantly greater than zero (*P* = 0.0045; greater annual production of *S*. *purpurea* above-ground biomass within than outside the exclosure), but the dynamic reversed beginning in 2011 (*β* = -114.56 g/m^2^/yr, *P* = 0.0005).

Because *S*. *purpurea* is the dominant perennial in the region and its response to livestock exclosure was complex, it is worth viewing in more detail. A typical, albeit not universal, pattern is shown for experiment 2 (Fig 8). In a number of experiments, production of *S*. *purpurea* often increased in the year following exclosure, but declined thereafter. Even in experiment 6, where mean abundance was greater post-exclosure than earlier, we noted an almost-significant decline after the first post-exclosure year (*β* = -7.44g/m^2^/yr, *t* = -2.015, *P* = 0.0564).

### Effects of reducing pika abundance

#### Biophysical variables

**Fig 9:**
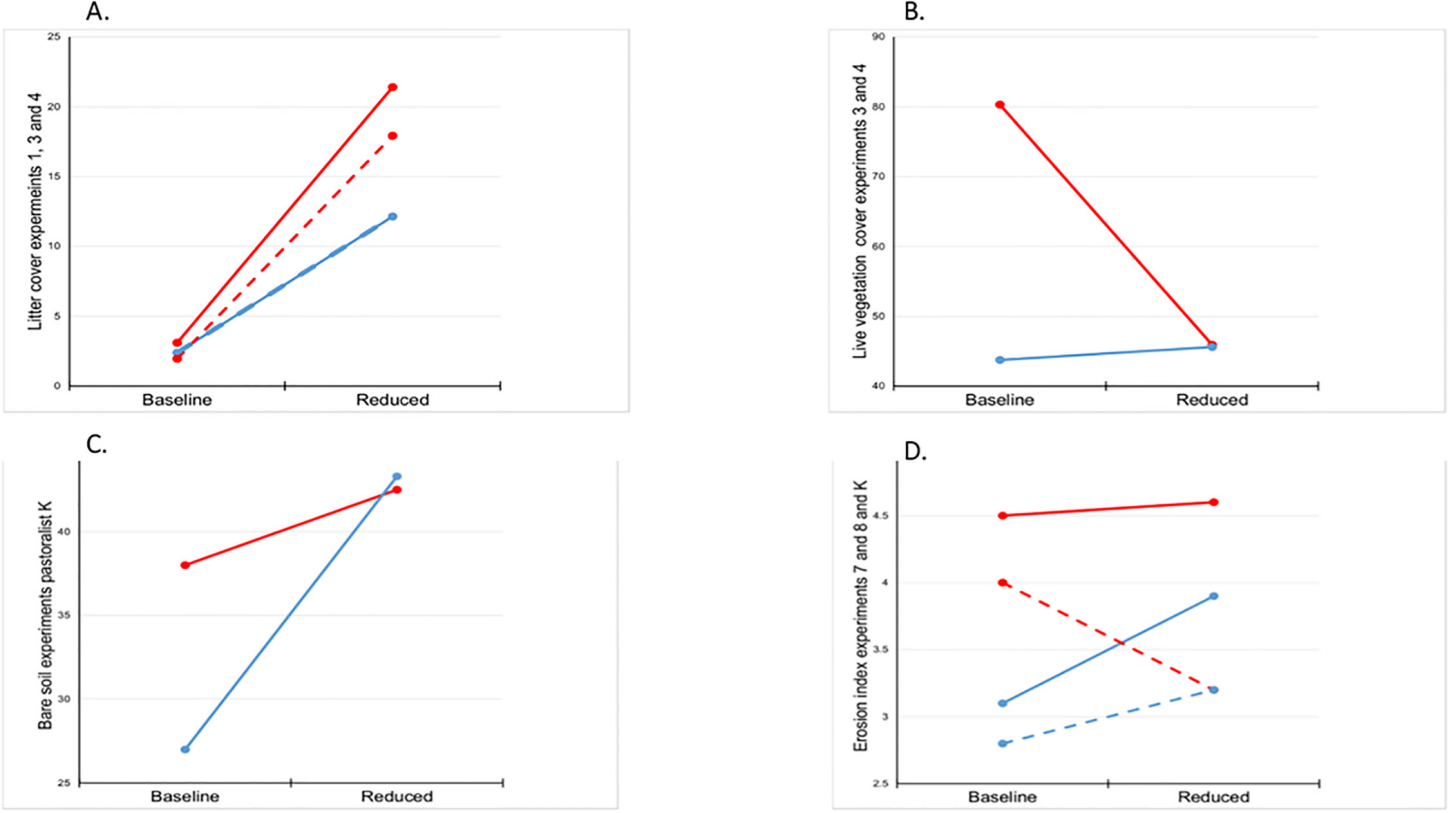
Patterns of biophysical variables where differences among comparable experiments were attributable to reductions in pika abundance (*P* < 0.025). Trends indicated by red lines are mean values from experiments in which pikas were reduced; blue lines from control experiments. A) litter cover, paired experiments 3 and 4 (solid lines), and experiments from pastoralist K (dashed lines); B) percent cover consisting of live vegetation, experiments 3 and 4; C) percent bare soil, experiments 1, 3, and 4; and D) erosion index, experiments 7 and 8 (solid lines), and experiments 1, 3, and 4 (dashed lines).

Reducing pika abundance resulted in increased accumulation of litter when comparing experiments 3 with 4, as well as 1 and 3 with 4 (Fig 9A, S1 Table, Statistical results corresponding with Fig 9), but not in other comparisons. However, this effect was evidently only when livestock were absent (2^nd^-order interaction with livestock exclosure; S1 Fig). Percent cover consisting of live vegetation declined more markedly in experiment 3 following pika reduction than in experiment 4 (Fig 9). Reducing pikas muted the increase of bare soil in experiments 1 and 3 relative to the pika-abundant experiment 4 (Fig 9C). Erosion indices increased less rapidly in experiment 7 after pika reduction than within the exclosure of the paired experiment 8 (Fig 9D, solid lines); in contrast, erosion increased in experiment 4, but declined within the exclosures of experiments 1 and 3 managed by the same pastoralist following pika reduction (Fig 9D, dashed lines).

#### Plant species

**Fig 10:**
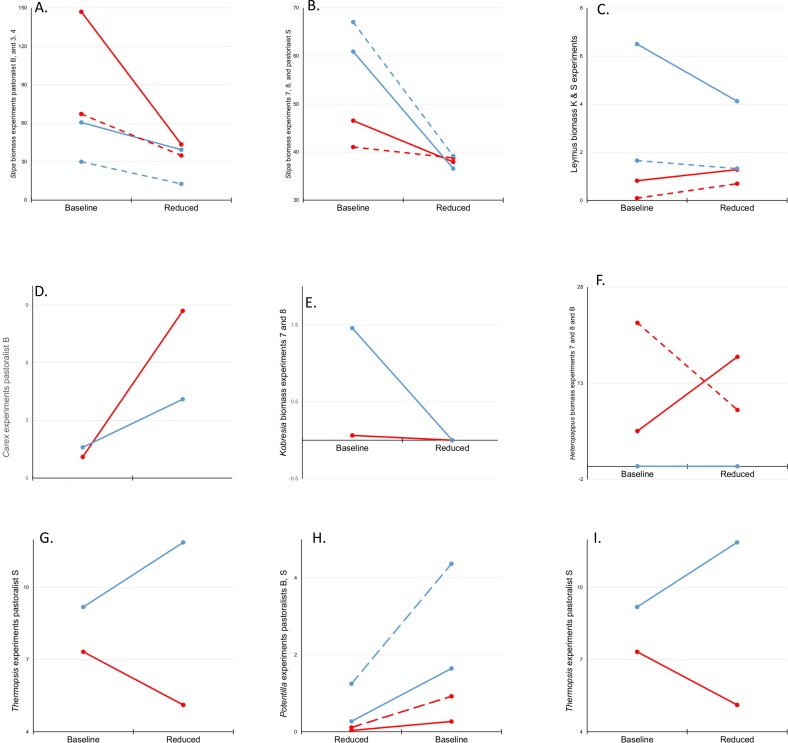
Patterns of vegetation biomass where differences among comparable experiments were attributable to reductions in pika abundance (interaction between experiment and period of pika reduction where implemented, *P* < 0.025). Trend indicated by red lines are mean values from experiments in which pikas were reduced; blue lines from control experiments. A) *Stipa purpurea*, experiments in pastures of Pastoralist B (solid lines) and experiments 3 and 4 (dashed lines); B) *S*. *purpurea* in experiments in pastures of Pastoralist S (solid lines), and experiments 7 and 8 (dashed lines); C) *Leymus secalinus*, experiments in pastures of Pastoralist K (solid lines), and Pastoralist S (dashed lines); D) *Carex* spp., experiments 2 and 5; E) *Kobresia* spp., experiments 7 and 8; F) *Heteropappus altaicus*, experiments in pastures of Pastoralist B (solid lines), and 7 and 8 (dashed line); G) *Potentilla bifurca*, experiments 7 and 8 (solid lines) and in pastures of Pastoralist K (dashed lines); H) *P*. *bifurca*, experiments in pastures of Pastoralist B (solid lines), and Pastoralist S (dashed lines); I) *Thermopsis lanceolata*, experiments in pastures of Pastoralist S.

Reducing pikas produced significant changes in annual production of *S*. *purpurea* in all but 2 experiments (numbers 2 and 12), but the patterns were inconsistent (S2 Table, Statistical results corresponding with Fig 10). *S*. *purpurea* declined generally between the pre- and post-reduction periods, and this decline (greater with livestock excluded, S1 Fig) was exacerbated by reducing pikas in experiments 2 and 3 relative to their controls (Fig 10A), as well as experiment 3 relative to 4. However, reducing pikas evidently tempered the decline in *S*. *purpurea* in most other experiments (Fig 10B; S1 Fig). *L*. *secalinus* decreased in experiment 4 while it increased in its pair (experiment 3; Fig 10C) following pika reduction; a similar pattern was observed in experiments on pastures controlled by S (Fig 10C). Similarly, *Carex* spp. increased more in experiment 2 following pika reduction than its paired experiment 5 (Fig 10D). Annual production of *Kobresia* spp. did not change after pika reduction in experiment 7, but it declined markedly in its control pair, experiment 8 (Fig 10E).

Effects of pika reduction on the remaining common species were inconsistent. *H*. *altaicus* remained rare but unchanged in control experiments 8 and 5, while annual production declined with pika reduction in experiment 7 (dashed line, Fig 10F) but increased in experiment 2 (solid line, Fig 10IF, S1 Fig). Pika reduction accompanied an increase in annual production of *P*. *bifurca* in experiment 7 compared with 8, as well as in K’s pastures (Fig 10G), but pika reduction tempered increases in *P*. *bifurca* production in the 3 other comparisons (Fig 10H). Reducing pikas was associated with a reduction *in T*. *lanceolata* within experiments controlled by S, while it increased in experiments without pika reduction (Fig 10I).

## Discussion

### Effects of excluding livestock

As expected because of varied site history as well as differential intensity of herbivory during the study period, responses to livestock exclusion varied by experiment. Few responses to livestock exclosure were consistently evident among all 12 experiments (Table 5). The short duration of our study (4 years following exclosure) may also have precluded us from observing phenomena that require longer to develop. That said, the 43 significant (*P* < 0.025) responses (from a total possible 156 comparisons) suggest patterns that appear to us robust and interpretable. Note also that many species-level comparisons failed to exhibit livestock effects due to the species’ rarity both within and outside our exclosures (Table 2).

**Table 5:** Summary of the effect of livestock presence on quantifiable response variables, by livestock exclosure experiments.

Response/Experiment	1	2	3	4	5	6	7	8	9	10	11	12
Litter	-	-	-		-	-	-	-	-	-	-	
Bare soil	+	+			+	+						
Erosion	+		+		+	+		+	+			+
Live cover	-	-	+								+	+
*Cardamine*	-/+	+								+		
*Carex*												
*Heteropappus*							+		-/+	+	+	
*Kobresia*		+			+							
*Leymus*												
*Oxytropis*			-	-								
*Poa*												
*Potentilla*				+							+	
*Stipa*		-/+			-	-/+						-/+
*Thermopsis*			+	+				-/+				

Cell entries marked with by ‘+’ symbols represent significant increases (*P* < 0.025) with presence of livestock on the variable (percent cover for litter, bare soil, and live cover; index value for erosion; proportional change in annual production for plant species); negative (-) symbols represent significant declines with livestock presence, either as a categorical response or trend with time. The notation (-/+) indicates an initial negative response, followed later by a positive response. In this table, responses are to the presence of livestock, and thus directionality reverses those in statistical tests which examine effects of livestock exclusion. Table cells are blank where no significant effect was found.

Percent litter cover, unsurprisingly, was invariably higher within than outside exclosures. Litter measured during the summer following winter-time grazing simply represented vegetation remaining on the ground rather than consumed by livestock herbivores. Our field protocol did not allow quantifying the biomass represented by this litter, and thus we lacked a method to estimate consumption rate of fresh biomass by livestock. To conclude that livestock negatively affected litter abundance is simply to reiterate the obvious: vegetation not consumed dies during winter and some remains as litter the subsequent summer. However, both our qualitative index of erosion and percent bare soil showed consistent responses to short-term protection from herbivory and attendant trampling. We found no cases in which bare soil or erosion indices increased with protection from livestock, and found significant improvements in 11 of 24 comparisons.

In line with part of our expectation, herbivory and trampling appeared beneficial to the competitive ability of species rarely consumed by sheep. Annual production of herbaceous forbs such as *Cardamine*, *Heteropappus*, *Potentilla*, and *Thermopsis* all declined within exclosures relative to their abundances under grazing (Figs 6 and 7). Thus, grazing and trampling appeared to promote the ability of these species to compete with graminoids.

However, contrary to our other expectation, we found little evidence that livestock, at the densities and intensities in our study, reduced annual productivity of species preferred as forage. We found no patterns with livestock exclosure on annual production of either the frequently consumed sedge *Carex*, or of the preferred grasses *L*. *secalinus* or *Poa* (although the rarity of all 3 compromised our power to detect any dynamics; Table 2). In most cases, herbivory had no demonstrated effect on annual production of the mat-like *Kobresia*, and in 2 experiments excluding livestock reduced their productivity. Thus our results supported those of Miehe [44–46], who had previously suggested that *Kobresia* spp. in many alpine associations in central Tibet were resistant to and adapted to herbivory.

**Fig 11:**
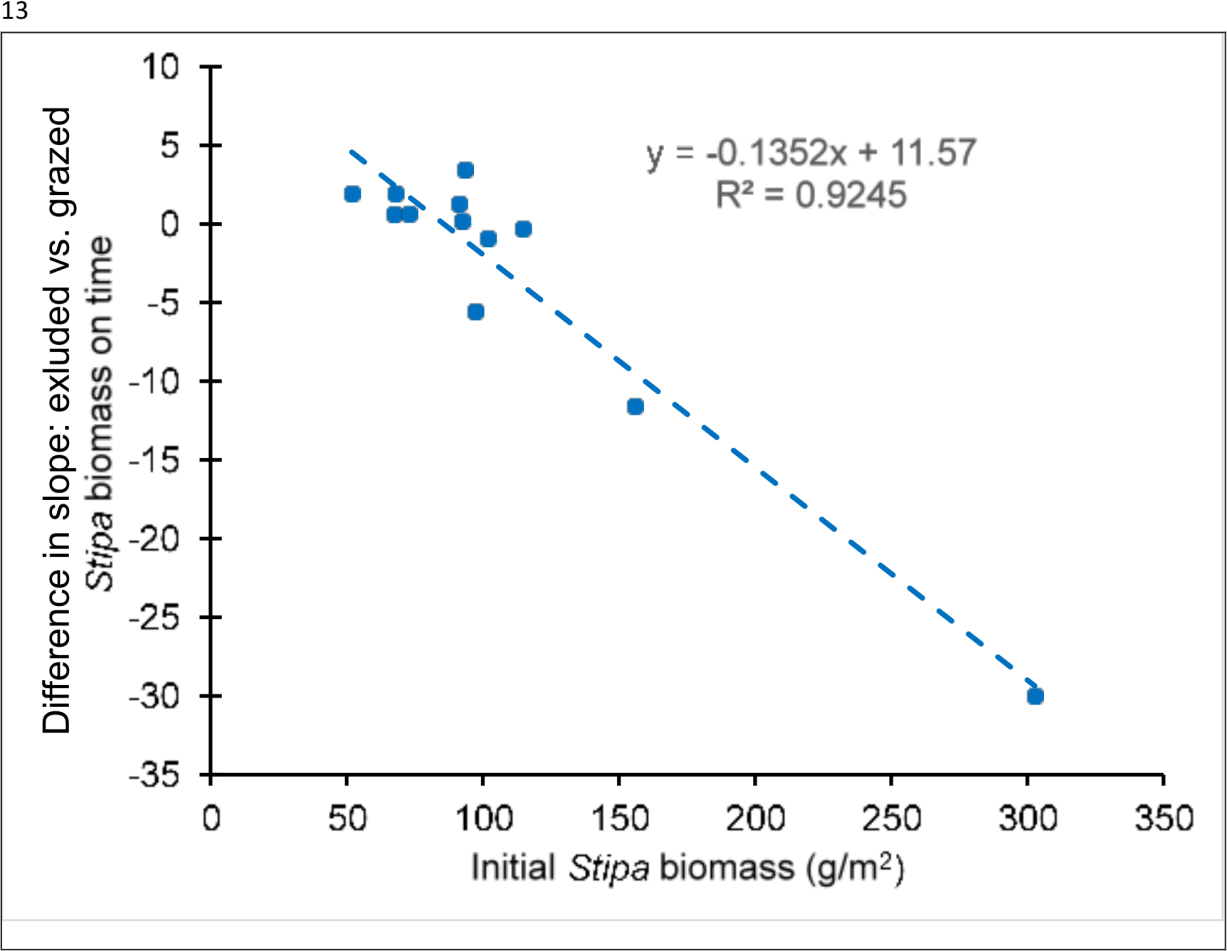
Relationship of differences between excluded and grazed plots in annual rate of change of *S*. *purpurea* biomass production (2009–2013) with its initial abundance in 2009. Shown are paired, mean differences of slopes on time.

In only 1 experiment did we find that protection from livestock increased the annual production of the dominant (and preferred) grass, *S*. *purpurea*. More commonly, we found a pattern in which *S*. *purpurea* increased in the initial year following exclosure, but declined subsequently with protection, most often to below its initial productivity (Figs 7 and 9). Our observations (Fig 3) suggest that shading or competition for below-ground resources by the dominant species, *S*. *purpurea* exerted a negative feedback effect on its ability to generate new production. This result is further supported by the finding that, across all experiments, although *S*. *purpurea* within livestock exclosures declined with time (*β* = -8.669, SE = 1.035, *t* = -8.38, *P* < 0.0001), the rate of decline was itself strongly negatively associated with its own abundance within the exclosure at the experiment’s initiation (year × initial biomass interaction *β* = -0.192, SE = 0.016, *t* = -11.83, *P* < 0.0001, *n* = 359), and this negative density-dependence was much stronger in excluded than grazed experiments (Fig 11). Interestingly, we found no similar dynamic operating in *T*. *lanceolata*, consistent with our observations that, under appropriate conditions, it tends to form dense monocultures.

Curiously, one unpalatable group of species that we expected to decline with protection from grazing relative to grazed plots, *Oxytropis*, displayed the opposite trend. We note, however, that it was present in more than trace amounts in only 1 of the 12 experiments, and may have begun declining toward the end of the study (Fig 7). Thus although intriguing, we hesitate to speculate further on why the response of *Oxytropis* confounded our expectation.

It is worth noting that we lacked power to examine trends in *Poa* spp. because it was so rarely encountered in our plots. Future studies might profitably focus on this genus, because its prevalence in sheep diets (combined with its rarity on the landscape) suggests it is highly selected by sheep. Thus, whether *Poa* species decline with herbivory, or conversely display a tolerance similar to those documented here, could be an important determinant of the overall sustainability of these grazing systems.

These responses to exclusion suggest that common forage species in this area were well adapted to winter-time grazing. In most cases, the additional competition for resources that followed grazing exclusion evidently constrained the ability of plants to produce above-ground biomass that more than compensated any limitation produced by removal of the previous years’ growth. The contrasting patterns of livestock simultaneously generating more bare patches and erosion, while facilitating regrowth among most species, led to inconsistent and conflicting trends in overall percent of ground covered by live vegetation. We found that protection from livestock increased percent cover of live vegetation in 3 experiments, decreased it in 3 experiments, and had no discernible effect in 6 experiments.

### Effects of reducing pikas

As in the case of the more straightforward analysis of livestock exclosure, experiments varied in evidence of response to pika reduction. Independent of the effects of excluding livestock, higher abundance of pikas reduced litter, and increased bare soil and indices of erosion relative to lower abundances in some, but not all, comparisons (Table 6). Unlike livestock which migrated to summer pastures, pikas were resident (and consuming vegetation) on experiments year-round.

**Table 6:** Summary of the effect of pikas on quantifiable response variables.

Response/Experiment	*3* v 4	*7* v 8	*K*	*B*	*S*
Litter	-		-		
Bare soil			+		
Erosion		+	+		
Live cover	+				
Cardamine					
Carex				-	
Heteropappus		+		-	
Kobresia		-			
Leymus			-		-
Oxytropis					
Poa					
Potentilla		-	-	+	+
Stipa	+	-		+	-
Thermopsis					+

Compared are livestock exclosure experiments with and without pika reduction, 2009–2013, Village Five, Gouli Township, Dulan County, Qinghai Province, China. Positive responses (indicated by +) symbols represent significantly positive (P < 0.025) effects of pika presence on the variable; negative (-) symbols represent significantly negative effects of pika presence on the variable. Thus as illustrated in this table, responses are the reverse of those presented in the text, which describe mixed-effect models testing effects of pika reduction. Table cells are blank where no significant effect was found. Pikas were reduced in experiments denoted by italic font; pikas were uncontrolled in experiments denoted by bold font.

Reducing pika densities had mixed, and at times contradictory effects on annual production of vegetation. In some cases, pikas at their uncontrolled densities appeared to promote the production of forbs, such as *Potentilla*, while limiting production of their preferred forage, sedges. In some cases, pikas at uncontrolled densities appeared to restrict production of *Stipa*, in others to promote it. In all cases, the relative magnitude of changes were modest.

Most investigators who have examined pika-grassland dynamics have concluded, contrary to earlier assertions [8], that high pika densities are more likely a response to low vegetation density than its cause [4,10]). A more nuanced view would suggest the following: Although in most cases pika abundance has little effect, under some combinations of circumstances their foraging, clipping and burrowing activities can perpetuate conditions that reduce rangeland value to pastoralists (such as high proportion of bare soil, low vegetation cover, and persistence of otherwise poorly-competing plant species) whose ultimate causes lie elsewhere. Our pika reduction experiments lend support to this view. In 3 of 10 comparisons, short-term reductions in pika abundance produced modest reductions in percent bare soil and indices of erosion relative to control plots. In 2 of 10 comparisons, increase in sedges may have resulted from reduced herbivory in pika-reduced areas. Effects of short-term pika reduction on the dominant, palatable graminoid, *S*. *purpurea* were inconsistent (in 2 cases positive, in 2 cases negative). We lack data on forage quality (e.g., protein content, digestibility), particularly as influenced by pikas that could be used to further explore the degree to which pikas compete with, or alternatively facilitate growth of livestock [68–69].

## Conclusions

Although we were only able to follow experiments for 4 years following treatments and efforts to reduce pika densities were less effective than initially intended (Table 3), our livestock exclusion did have an appreciable effect, as was clearly evident on simple inspection. However, that our livestock exclusion had an appreciable effect, at least superficially, was clearly evident on simple inspection. After only one-and-a-half grazing winters, 11 of the 12 fenced-exclosures were clearly visible in an image appearing on Google earth taken on 6 December 2010. Our high threshold for statistical significance may have prevented us from recognizing subtle, yet real effects. Physical effects of grazing by both livestock and, in some cases, pikas were readily detectable within the few years’ of establishment of our exclosures. Litter, percent bare soil, and indices of erosion responded in uniform and predictable (if not always statistically significant) ways to both relief from livestock grazing and reduction in pika abundance.

We initially hypothesized that we would detect differences in the response of plant species that reflected their degree of adaptation to and tolerance of herbivory [70]. We found little evidence that species present in sufficient abundance to examine differed from one another in their response to livestock presence (Table 5) or pika abundance (Table 6), at least at the grazing intensities [71] encountered and levels of pika reduction achieved. We suspect that most plant species capable of persisting at appreciable densities while exposed to continuous herbivory have co-evolved to tolerate, and perhaps benefit from grazing. Species that decline in the presence of substantial herbivory were probably long ago either relegated to small refugia where our sampling failed to detect them, present in such low abundance that we lacked power to identify responses, or completely absent from the area. Our data limit our ability to speculate on the relative importance that compensatory growth, shading, associational defense, below-ground competition for nutrients or water, soil legacies, nutrient deposition from dung and urine, physical alterations, or other interactions have had in prompting regrowth of most Tibetan steppe species exposed to winter-season grazing at these or similar intensities [47, 72–77]. We also caution that the dynamics we observed might differ in systems exposed to more intense grazing pressure [78], and/or grazing during the growing season. The estimated grazing pressure in our study area (0.05ha^-1^ to 0.36 ha^-1^ sheep equivalents) was lower than the 0.16 ha^-1^ to 2.05 ha^-1^ reported from other recent exclosure studies on the QTP [48,54,56,58].

Recent Chinese policy has emphasized restoration via long-term grazing exclusion [48–59]. Our work suggests that physical manifestations of unsustainable use, such as bare soil, rills, gulleys, and pedestalling, may respond positively to short-term relief from grazing. However, claims that vegetation in QTP steppe systems requires complete rest (or pika removal) to achieve greater productivity should be reconsidered in light of these results. Although grazing, trampling, and herbivory from wildlife no doubt exert negative effects when sufficiently intense, these QTP steppe plants appear well adapted to moderate levels of offtake and disturbance.

## Supporting Information

S1 Fig Where pika and livestock effects exhibited significant interaction, pika effects both within (left-hand panel of each pair) and outside (right-hand panel of each pair) of fenced livestock exclosures are shown. As in text, red lines display mean values from experiments in which pikas were reduced, whereas blue lines diplay pattners of abundance during the same time period from experiments in which pikas remained uncontrolled. In all cases, relationships within, outside of, and considering both together, were similar.
(DOCX)

S1 Table Statistical results corresponding with Fig 9. (DOCX)

S2 Table Statistical results corresponding with Fig 10. (DOCX)

## References

[1] Zhou HK, Zhao XQ, Tang YH, Gu S, Zhou L. Alpine grassland degradation and its control in the source region of Yangtze and Yellow rivers, China. Grassland Sci. 2005;51:191–203. Chinese, English abstract.

[2] Li XL, Gao J, Brierley G, Qiao YM, Zhang J, Yang YW. Rangeland degradation on the Qinghai-Tibet plateau: implications for rehabilitation. Land Degrad Dev. 2013;24:72–80.

[3] Shang ZH, Gibb MJ, Leiber F, Ismail M, Ding LM, Guo XS, Long RJ. The sustainable development of grassland-livestock systems on the Tibetan plateau: problems, strategies and prospects. Rangeland Journal. 2014;36:267–296.

[4] Holzner W, Kriechbaum M. Pastures in south and central Tibet (China)II. Probable causes of pasture degradation. Die Bodenkultur. 2001;52:37–44.

[5] Harris RB. Rangeland degradation on the Qinghai-Tibetan plateau: a review of the evidence of its magnitude and causes. J Arid Environ. 2010;74:1–12.

[6] Retzer V. Forage competition between livestock and Mongolian pika (*Ochotona pallasi*) in southern Mongolian mountain steppes. Basic Appl Ecol. 2007;8:147–157.

[7] Delibes-Mateos M, Smith AT, Slobodchikoff CN, Swenson JE. The paradox of keystone species persecuted as pests: a call for the conservation of abundant small mammals in their native range. Biol Conserv. 2011;144:1335–1346.

[8] Fan NC, Zhou WY, Wei WH, Wang QY, Jiang YJ. Rodent pest management in the Qinghai-Tibet alpine meadow ecosystem In: Singleton GR, Hinds LA, Leirs H, Zhang ZB, editors. Ecologically-based rodent management. Australian Centre for International Agricultural Research, Canberra; 1999 p. 285–304.

[9] Pech RP, Jiebu, Arthur AD, Zhang YM, Hui L. Population dynamics and responses to management of plateau pikas *Ochotona curzoniae* . J Appl Ecol. 2007;44:615–624.

[10] Shi YZ. On the influence of rangeland vegetation to the density of plateau pika (*Ochotona curzoniae*). Acta Theriologica Sinica. 1983;3:181–187. Chinese, English abstract.

[11] Qu JP, Li WJ, Yang M, Ji WH, Zhang YM. Life history of the plateau pika (*Ochotona curzoniae*) in alpine meadows of the Tibetan plateau. Mamm Biol. 2013;78:68–72.

[12] Schaller GB. Wildlife of the Tibetan steppe. Chicago: University of Chicago Press; 1998.

[13] Harris RB. Wildlife conservation in China: preserving the habitat of China’s wild west. Armonk, NY: M.E. Sharpe, Inc.; 2008.

[14] Wangdwei M, Steele B, Harris RB. Demographic responses of plateau pikas to vegetation cover and land use in the Tibetan Autonomous Region, China. J Mammal. 2013;94:1077–1086.

[15] Smith AT, Formozov NA, Hoffmann RS, Zheng CL, Erbajeva MA. The pikas In: Chapman JA, Flux JEC, editors. Rabbits, hares, and pikas: status survey and conservation action plan. Gland, Switzerland:IUCN; 1990 p. 14–60.

[16] Smith AT, Johnston CH. *Ochotona curzoniae* IUCN Red List of Threatened Species. Version 2013.2. [cited 5 April 2014]. Available: www.iucnredlist.org.

[17] Smith AT, Zahler P, Hinds LA. Ineffective and unsustainable poisoning of native small mammals in temperate Asia: a classic case of the science-policy divide In: McNeely JA, McCarthy TM, Smith AT, Olsvig-Whittaker L, Wikramanayake ED, editors. Conservation biology in Asia. Kathmandu: Society for Conservation Biology and Resources Himalaya Foundation; 2006 p. 285–293.

[18] Smith AT, Foggin JM. The plateau pika as a keystone species for biodiversity on the Tibetan plateau. Anim Conserv. 1999;2:235–240.

[19] Lai CH, Smith AT. Keystone status of plateau pika (*Ochotona curzoniae*): effect of control on biodiversity of native birds. Biodivers Conserv. 2003;12:1901–1912.

[20] Xu AC, Jiang ZG, Li CW, Guo JX, Wu GS, Cai P. Summer food habits of brown bears in Kekexili Nature Reserve, Qinghai-Tibetan plateau, China. Ursus. 2006;17:132–137.

[21] Arthur AD, Pech RP, Davey C, Jiebu, Zhang YM, Lin H. Livestock grazing, plateau pikas and the conservation of avian biodiversity on the Tibetan plateau. Biol Conserv. 2008;141:1972–1981.

[22] Hogan BW. The plateau pika: a keystone engineer on the Tibetan plateau [Ph.D. dissertation]. Tempe (AZ): Arizona State University; 2010.

[23] Harris RB, Zhou JK, Ji YQ, Zhang K, Yang YH, Yu D. Evidence that the Tibetan fox is an obligate predator of the plateau pika: conservation implications. J Mammal. 2014;95: 1207–1221.

[24] Wilson MC, Smith AT. The pika and the watershed: the impact of small mammal poisoning on the ecohydrology of the Qinghai-Tibetan plateau. Ambio. 2015;44:16–22. 10.1007/s13280-014-0568-x 25331028 PMC4293360

[25] Frauenfeld O, Zhang TJ, Serreze MC. Climate change and variability using European Centre for Medium-Range Forecasts reanalysis (ERA-40) temperature on the Tibetan plateau. J Geophys Res. 2005;110:D02101, 10.1029/2004JD005230

[26] Chen SB, Liu YF, Thomas A. Climatic change on the Tibetan plateau: potential evapotranspiration trends from 1961–2000. Clim Change. 2006;76:291–319.

[27] Zhao L, Ping CL, Yang DQ, Cheng GD, Ding YJ, Liu SY. Changes of climate and seasonally frozen ground over the past 30 years in Qinghai-Xizang (Tibetan) Plateau, China. Glob Planetary Change. 2004;43:19–31.

[28] Gao YH, Cuo L, Zhang YX. Changes in moisture flux over the Tibetan plateau during 1979–2011 and possible mechanism. J Climate. 2014; 10.1175/JCLI-D-13-00321.1

[29] Zhang YQ, Welker JM. Tibetan alpine tundra responses to simulated changes in climate: aboveground biomass and community responses. Arct Antarct Alp Res. 1996;28:203–209.

[30] Yu H, Luedeling E, Xu J. Winter and spring warming result in delayed spring phenology on the Tibetan plateau. Proc Natl Acad Sci USA. 2010;107:22151–22156. 10.1073/pnas.1012490107 21115833 PMC3009774

[31] Shen M. Spring phenology was not consistently related to winter warming on the Tibetan plateau. Proc Natl Acad Sci USA. 2011;108:E91–E92. 10.1073/pnas.1018390108 21482816 PMC3093521

[32] Yi S, Zhou Z. Increasing contamination might have delayed spring phenology on the Tibetan plateau. Proc Natl Acad Sci USA. 2011;108:E94 10.1073/pnas.1100394108 21482817 PMC3093522

[33] Piao SL, Cui MD, Chen AP, Wang XH, Ciais P, Liu J, et al Altitude and temperature dependence of change in the spring vegetation green-up date from 1982 to 2006 in the Qinghai-Xizang Plateau. Agric For Meteorol. 2011;151:1599–1608.

[34] Shen MG, Tang YH, Chen J, Zhu XL, Zheng YH. Influences of temperature and precipitation before the growing season on spring phenology in grasslands of the central and eastern Qinghai-Tibetan plateau. Agric For Meteorol. 2011;151:1711–1722.

[35] Chen H, Zhu Q, Wu N, Wang Y, Peng CH. Delayed spring phenology on the Tibetan plateau may also be attributable to other factors than winter and spring warming. Proc Natl Acad Sci USA. 2011;108:E93 10.1073/pnas.1100091108 21482815 PMC3093455

[36] Luedeling E, Yu HY, Xu JC. Replies to Shen, Chen et al., and Yi and Zhou: linear regression analysis misses effects of winter temperature on Tibetan vegetation. Proc Natl Acad Sci USA. 2011;108:E95.

[37] Yu HY, Xu JC, Okuto E, Luedeling E. Seasonal response of grasslands to climate change on the Tibetan plateau. PLoS ONE. 2012;7(11):e49230, 10.1371/journal.pone.0049230 23173048 PMC3500274

[38] Wu N, Yan ZL. Climate variability and social vulnerability on the Tibetan plateau: Dilemmas on the road to pastoral reform. Erdkunde. 2002;56:2–14.

[39] Cao JJ, Yeh ET, Holden NM, Yang YY, Du GZ. The effects of enclosures and land-use contracts on rangeland degradation on the Qinghai-Tibetan plateau. J Arid Environ. 2013;97:3–8.

[40] Ryavec KE, Veregin H. Population and rangelands in Central Tibet: a GIS-based approach. GeoJournal. 1998;44 1:61–72.

[41] Wang LW, Wei YX, Niu Z. Spatial and temporal variations of vegetation in Qinghai Province based on satellite data. J Geogr Sci. 2008;18:73–84. 18763535

[42] Wang XD, Zhong XH, Liu SZ, Liu JG, Wang ZY, Li MH. Regional assessment of environmental vulnerability in the Tibetan plateau: development and application of a new method. J Arid Environ. 2008;72:1929–1939.

[43] Yang YH, Fang JY, Pan YD, Ji CJ. Aboveground biomass in Tibetan grasslands. J Arid Environ. 2008;73:91–95.

[44] Miehe GS, Meihe S, Kaiser K, Liu JQ, Zhao XQ. Status and dynamics of the *Kobresia pygmaea* ecosystem on the Tibetan plateau. Ambio. 2008;37:272–279. 18686506 10.1579/0044-7447(2008)37[272:sadotk]2.0.co;2

[45] Miehe G, Miehe S, Bach K, Nölling J, Hanspach J, Reudenbach C, et al Plant communities of central Tibetan pastures in the alpine steppe/*Kobresia pygmaea* ecotone. J Arid Environ. 2011;75:711–723.

[46] Miehe G, Meihe S, Böhner J, Kaier K, Hensen I, Madsen D, et al How old is the human footprint in the world’s largest alpine ecosystem? A review of multiproxy records from the Tibetan plateau from the ecologists’ viewpoint. Quat Sci Rev. 2014; 86:190–209.

[47] Graff PM, Aguiar A, Chaneton EJ. Shifts in positive and negative plant interactions along a grazing intensity gradient. Ecology. 2007; 88:188–199. 17489467 10.1890/0012-9658(2007)88[188:sipanp]2.0.co;2

[48] Wu GL, Du GZ, Liu ZH, Thirgood S. Effect of fencing and grazing on a *Kobresia*-dominated meadow in the Qinghai-Tibetan plateau. Plant Soil. 2009;319:115–126.

[49] Dong SK, Li JP, Li XY. Application of design theory for restoring the “black beach” degraded rangeland at the headwater areas of the Qinghai-Tibetan Plateau. Afr J Agric Res 2010; 5:3542–3552.

[50] Gao YH, Zeng XY, Schumann M, Chen H. Effectiveness of exclosures on restoration of degraded alpine meadow in the eastern Tibetan plateau. Arid Land Res Manage. 2011;25:164–175.

[51] Wei D, Xu R, Wang YH, Wang YS, Liu YW, Yao TD. Responses of CO_2_, CH_4_ and N_2_O fluxes to livestock exclosure in an alpine steppe on the Tibetan plateau, China. Plant Soil. 2012;359:45–55.

[52] Wu JS, Zhang XZ, Shen ZX, Shi PL, Yu CQ, Song MH, et al Species richness and diversity of alpine grasslands on the northern Tibetan plateau: effects of grazing exclusion and growing season precipitation. J Resour Ecol. 2012;3:236–242.

[53] Li YY, Dong SK, Wen L, Wang XX, Wu Y. The effects of fencing on carbon stocks in the degraded alpine grasslands of the Qinghai-Tibetan Plateau. J. Environ Manage. 2013;128: 393–399. 10.1016/j.jenvman.2013.05.058 23792816

[54] Shi XM, Li XG, Li CT, Zhao Y, Shang ZH, Ma QF. Grazing exclusion decreases soil organic C storage at an alpine grassland of the Qinghai-Tibetan plateau. Ecol Eng. 2013;57:183–187.

[55] Luan JW, Cui LJ, Xiang CH, Wu JH, Song HT, Ma QF, et al Different grazing removal exclosures effects on soil C stocks among alpine ecosystems in east Qinghai-Tibet plateau. Ecol Eng. 2014;64:262–268.

[56] Shang ZH, Deng B, Ding LM, Ren GH, Xin GS, Liu ZY, et al The effects of three years of fencing enclosure on soil seed banks and the relationship with above-ground vegetation of degraded alpine grasslands of the Tibetan plateau. Plant Soil. 2013;364:229–244.

[57] Sun J, Wang XD, Cheng GW, Wu JB, Hong JT, Niu SL. Effects of grazing regimes on plant traits and soil nutrients in an alpine steppe, northern Tibetan plateau. PLoS ONE. 2014;9(9):e108821, 10.1371/journal.pone.0108821 25268517 PMC4182571

[58] Wu JS, Zhang XZ, Shen ZX, Shi PL, Xu XL, Li XJ. Grazing-exclusion effects on aboveground biomass and water-use efficiency of alpine grasslands on the northern Tibetan plateau. Range Ecol Manage. 2013;66:454–461.

[59] Wu JS, Shen XZ, Shi PL, Zhou YT, Zhang XZ. Effects of grazing exclusion on plant functional group diversity of alpine grasslands along a precipitation gradient on the northern Tibetan plateau. Arct Antarct Alp Res. 2014;46:419–429.

[60] Harris RB, Bedunah DJ, Yeh EY, Smith AT, Anderies JM. Determinants of rangeland dynamics on the Qinghai-Tibet plateau, China: livestock, wildlife, and pastoralism. Pastoralism. 2010;1:325–326.

[61] Yeh ET, Gaerrang. Tibetan pastoralism in neoliberalising China: continuity and change in Gouli. Area. 2011;43:165–172. 10.1111/j.1475-4762.2010.00976.x

[62] Hutchinson MF. Interpolating mean rainfall using thin plate smoothing splines. Int J Geogr Inf Sci. 2001;9:385–403.

[63] Hutchinson MF. ANUSPLIN version 4.2 User Guide. Canberra: Centre for Resource and Environmental Studies, Australian National University; 2001.

[64] Hutchinson MF, McKenney DW, Lawrence K, Pedlar JH, Hopkinson RF, Milewska E, et al Development and testing of Canada-wide interpolated spatial models of daily minimum-maximum temperature and precipitation for 1961–2003. J Appl Meteor Clim. 2009;48:725–741.

[65] Liu YS. International hunting and the involvement of local people, Dulan, Qinghai, People’s Republic of China In: Bissonette JA, Krausman PR, editors. Integrating people and wildlife for a sustainable future. Bethesda: The Wildlife Society; 1995 p. 63–67.

[66] Zhang YM, Liu JK, Zhang ZB. Burrowing rodents as ecosystem engineers: the ecology and management of plateau zokors *Myospalax fontanierii* in alpine meadow ecosystems on the Tibetan plateau. Mamm Rev. 2003;33:284–294.

[67] National Research Council (NRC) Committee on Rangeland Classification. Rangeland health: new methods to classify, inventory, and monitor rangelands Washington, DC: National Academies Press; 1994.

[68] Augustine DJ, Springer TL. Competition and facilitation between a native and a domestic herbivore: trade-offs between forage quantity and quality. Ecol Appl. 2013;23:850–863. 23865235 10.1890/12-0890.1

[69] Detling JK. Do prairie dogs compete with livestock? In: Hoogland JL, editor. Conservation of the black-tailed prairie dog. Washington DC: Island Press; 2006 p. 65–88.

[70] Del-Val E, Crawley MJ. Are grazing increaser species better tolerators than decreasers? An experimental assessment of defoliation tolerance in eight British grassland species. J Ecol. 2005;93:1005–1016.

[71] Yang ZL, Guo H, Zhang JY, Du GZ. Stochastic and deterministic processes together determine alpine meadow plant community composition on the Tibetan plateau. Oecologia. 2013;171:495–504. 10.1007/s00442-012-2433-6 22923076

[72] Hilbert DW, Swift DM, Detling JK, Dyer MI. Relative growth rates and the grazing optimization hypothesis. Oecologia. 1981;51:14–18. 28310302 10.1007/BF00344645

[73] McNaughton SJ. Compensatory plant growth as a response to herbivory. Oikos. 1983;40:329–336.

[74] Cingolani AM, Posse G, Collantes MB. Plant functional traits, herbivore selectivity and response to sheep grazing in Patagonian steppe grasslands. J Appl Ecol. 2005;42:50–59.

[75] Ooesterheld M, Oyarzábal. Grass-to-grass protection from grazing in a semi-arid steppe. Facilitation, competition, and mass effect. Oikos. 2004;107:76–582.

[76] Hamilton EW, Frank DA. Can plants stimulate soil microbes and their own nutrient supply? Evidence from a grazing tolerant grass. Ecology. 2001;82:2397–2402.

[77] Hobbs NT. Modification of ecosystems by ungulates. J Wildl Manage. 1996;60:695–713.

[78] Yan L, Zhou GS, Zhang F. Effects of different grazing intensities on grassland production in China: a meta-analysis. PLoS ONE. 2013;8(12):e81466, 10.1371/journal.pone.0081466 24324694 PMC3855687

