# Supplementary material for: Herbivory and Competition of Tibetan Steppe Vegetation in Winter Pasture: Effects of Livestock Exclosure and Plateau Pika Reduction
Source: PLoS One. 2015 Jul 24;10(7):e0132897. doi: 10.1371/journal.pone.0132897 (PMC4514881; doi:10.1371/journal.pone.0132897)

**S1 Figure: Where pika and livestock effects exhibited significant interaction, pika effects both within (left-hand panel of each pair) and outside (right-hand panel of each pair) of fenced livestock exclosures are shown.** As in text, red lines display mean values from experiments in which pikas were reduced, whereas blue lines diplay pattners of abundance during the same time period from experiments in which pikas remained uncontrolled. In all cases, relationships within, outside of, and considering both together, were similar.

A. *Heteropappus altaica* biomass, experiments 2 and 5. Interaction of pika effect with exclosure effect: *β* = 1.26, SE = 0.45, *P* = 0.0063.


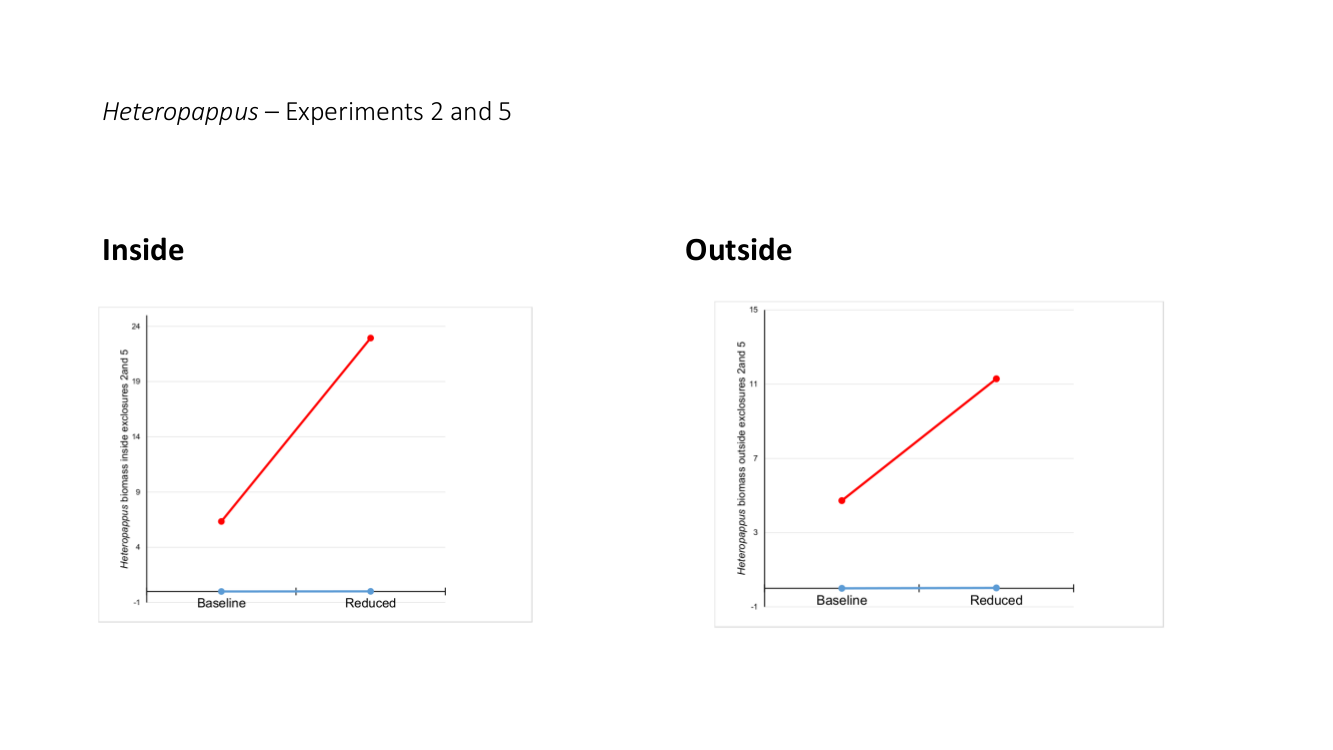


B. *Stipa purpurea* biomass, experiments 2 and 5. Interaction of pika effect with exclosure effect: *β* = -15.04, SE = 2.55, *P* < 0.0001.


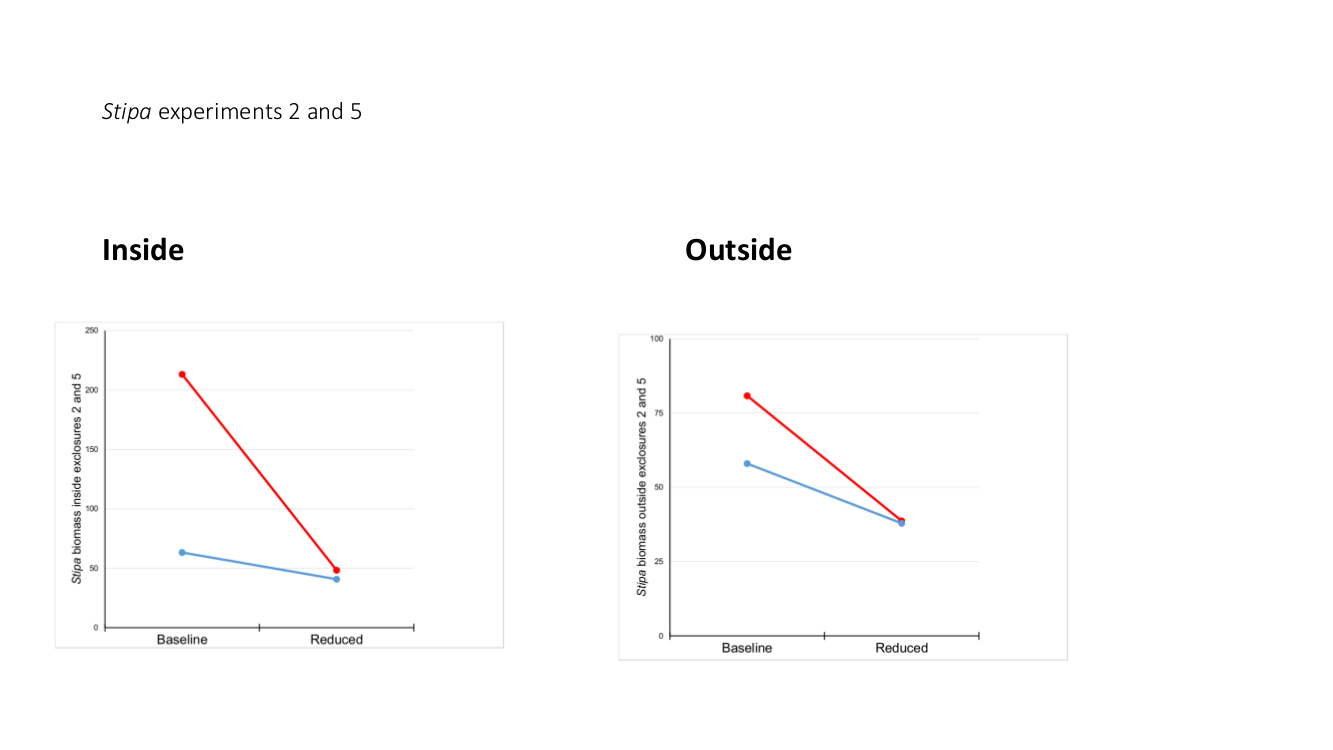


C. Litter cover (percent), experiments 3 and 4. Interaction of pika effect with exclosure effect: *β* = 1.72, SE = 0.62, *P* = 0.0066.
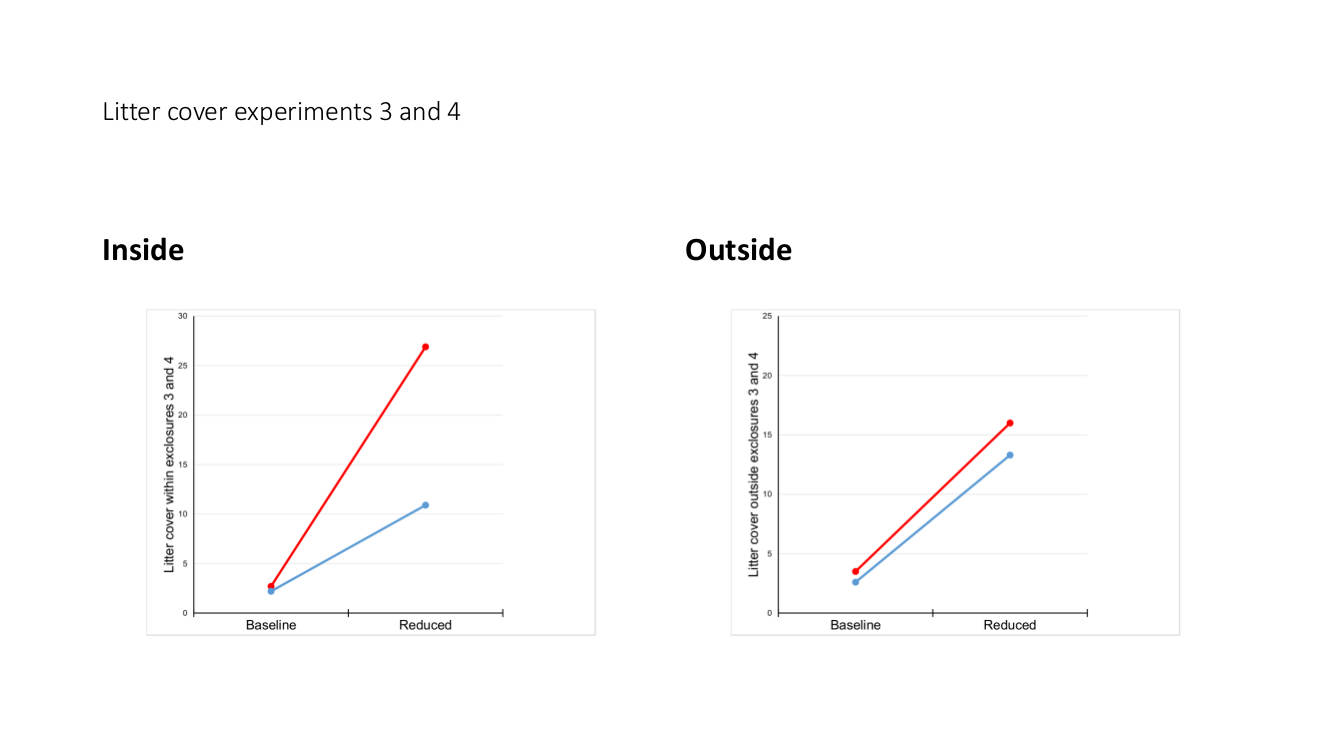


D. *Stipa purpurea* biomass, experiments 7 and 8. Interaction of pika effect with exclosure effect: *β* = 3.98, SE = 1.31, *P* = 0.0031.
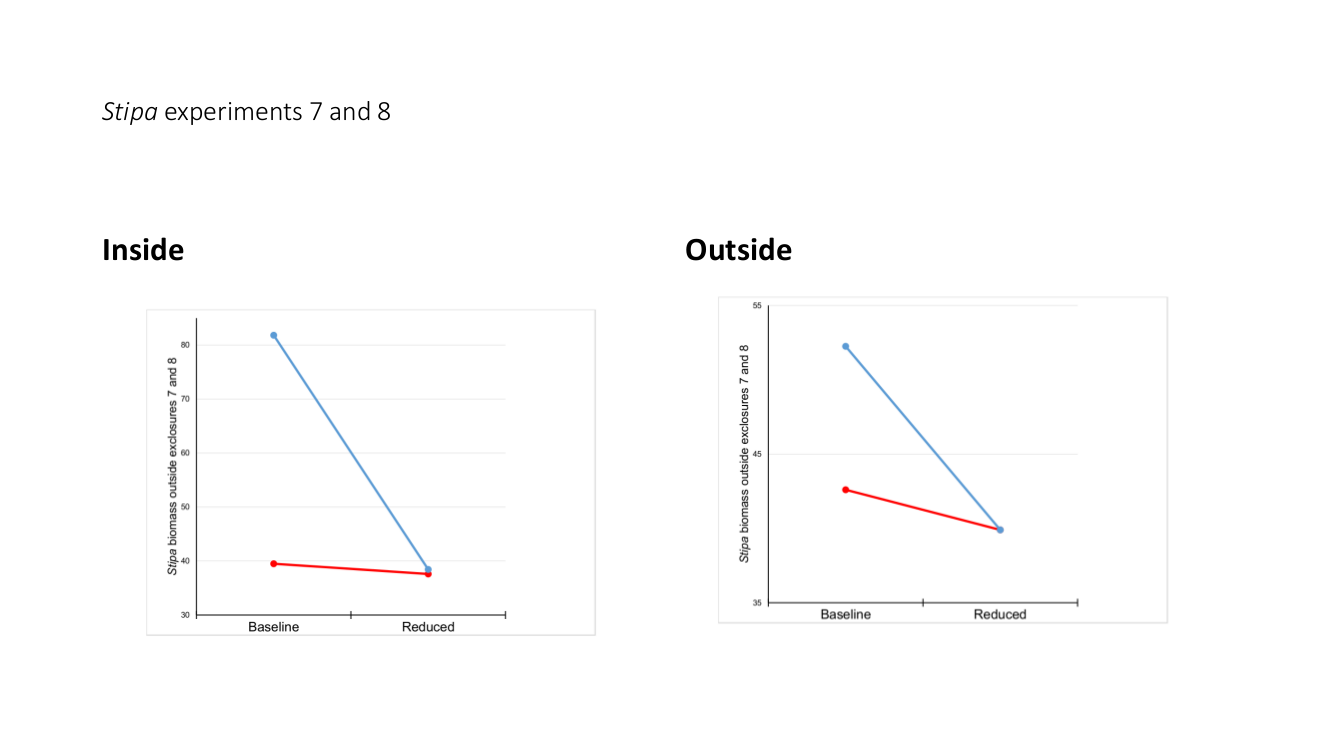

Supplement: S1 Fig — As in text, red lines display mean values from experiments in which pikas were reduced, whereas blue lines diplay pattners of abundance during the same time period from experiments in which pikas remained uncontrolled. In all cases, relationships within, outside of, and considering both together, were similar. (DOCX) [file pone.0132897.s001.docx]
