# Supplementary material for: Herbivory and Competition of Tibetan Steppe Vegetation in Winter Pasture: Effects of Livestock Exclosure and Plateau Pika Reduction
Source: PLoS One. 2015 Jul 24;10(7):e0132897. doi: 10.1371/journal.pone.0132897 (PMC4514881; doi:10.1371/journal.pone.0132897)
Supplement: S1 Table — (DOCX) [file pone.0132897.s002.docx]

**S1Table. Statistical results corresponding with Figure 9.**

| Figure | Experiments | Response | Interaction *β* | SE *(β)* | *F* | df | *P* |
| --- | --- | --- | --- | --- | --- | --- | --- |
| 9a | 3,4 | Litter | -2.168 | 0.62 | 12.13 | 1,92 | 0.0008 |
| 9a | K | Litter | 1.55 | 0.56 | 7.77 | 1,140 | 0.0060 |
| 9b | 3,4 | Live vegetation | 1.89 | 0.85 | 5.00 | 1,92 | 0.0277 |
| 9c | K | Bare soil | -2.94 | 1.09 | 7.26 | 1,140 | 0.0079 |
| 9d | 7,8 | Erosion index | -0.19 | 0.07 | 6.39 | 1,46 | 0.0150 |
| 9d | K | Erosion index | -0.31 | 0.10 | 9.21 | 1,70 | 0.0034 |

Shown for each figure are the experiments of interest, the response variable, the slope coefficient (*β*) of the interaction between pika treatment (reduced or not) and years during which treatment occurred (indicating an effect of pika reduction), the interaction coefficient standard error (SE), as well as the *F*, degrees of freedom (df) and probability value (*P*) of the interaction coefficient.
