# Supplementary material for: Herbivory and Competition of Tibetan Steppe Vegetation in Winter Pasture: Effects of Livestock Exclosure and Plateau Pika Reduction
Source: PLoS One. 2015 Jul 24;10(7):e0132897. doi: 10.1371/journal.pone.0132897 (PMC4514881; doi:10.1371/journal.pone.0132897)
Supplement: S2 Table — (DOCX) [file pone.0132897.s003.docx]

**S2 Table. Statistical results corresponding with Figure 10.**

| Figure | Experiments | Response | Interaction *β* | SE *(β)* | *F* | df | *P* |
| --- | --- | --- | --- | --- | --- | --- | --- |
| 10a | B | *S. purpurea* | 31.19 | 2.55 | 65.05 | 1,91 | <0.0001 |
| 10a | 3,4 | *S. purpurea* | 3.77 | 1.08 | 12.25 | 1,89 | 0.0007 |
| 10b | S | *S. purpurea* | 3.94 | 0.80 | 24.4 | 1,283 | <0.0001 |
| 10b | 7,8 | *S. purpurea* | -6.40 | 1.31 | 23.86 | 1,92 | <0.0001 |
| 10c | K | *L. secalinus* | 0.69 | 0.17 | 16.52 | 1,140 | <0.0001 |
| 10c | S | *L. secalinus* | 0.23 | 0.09 | 6.93 | 1,282 | 0.0089 |
| 10d | B | *Carex* | -0.64 | 0.16 | 15.19 | 1,92 | 0.0002 |
| 10e | 7,8 | *Kobresia* | 0.35 | 0.07 | 22.61 | 1,92 | <0.0001 |
| 10f | B | *H. altaica* | -2.89 | 0.45 | 41.44 | 1,91 | <0.0001 |
| 10f | 7,8 | *H. altaica* | 3.40 | 0.44 | 60.70 | 1,92 | <0.0001 |
| 10g | K | *P. bifurca* | -1.41 | 0.37 | 14.26 | 1,140 | 0.0002 |
| 10g | 7,8 | *P. bifurca* | -0.59 | 0.09 | 41.59 | 1,92 | <0.0001 |
| 10h | B | *P. bifurca* | -0.40 | 0.06 | 24.27 | 1,91 | <0.0001 |
| 10h | S | *P. bifurca* | -0.58 | 0.14 | 17.16 | 1,282 | <0.0001 |
| 10i | S | *T. lanceolata* | -1.22 | 0.40 | 9.49 | 1,282 | 0.0023 |

Shown for each figure are the experiments of interest, the response variable, the slope coefficient (*β*) of the interaction between pika treatment (reduced or not) and years during which treatment occurred (indicating an effect of pika reduction), the interaction coefficient standard error (SE), as well as the *F*, degrees of freedom (df) and probability value (*P*) of the interaction coefficient.
